# Kinetic Modeling of Iron Electrocoagulation in Moderate to Slightly Acidic Media

**DOI:** 10.1021/acsestwater.6c01166

**Published:** 2026-09-30

**Authors:** Edwin I. Ochedikwu, Akshat Verma, Shankararaman Chellam, Benjamin A. Wilhite, Jodie Lutkenhaus

**Affiliations:** † Artie McFerrin Department of Chemical Engineering, 14736 Texas A&M University, College Station, Texas 77843, United States; ‡ Department of Civil & Environmental Engineering, Texas A&M University, College Station, Texas 77843, United States; § Department of Materials Science and Engineering, Texas A&M University, College Station, Texas 77840, United States

**Keywords:** Electrocoagulation, kinetic modeling, water treatment, Fenton reaction, ferryl ion

## Abstract

Electrocoagulation is an emerging process for water and wastewater treatment. However, there is a gap in understanding the effects of electrocoagulation operation (i.e., pH, applied current, electrode configuration) and the concentration dependencies of reactive species involved in the degradation of contaminants. Here, we show the development and validation of a batch reactor kinetic model for an electrocoagulation process in moderate to slightly acidic media for water treatment, guided by experimental data of electrocoagulation in synthetic wastewater at pH values of 5.0, 5.5, 6.0, and 6.5. The kinetic model uniquely predicts the full transient profile of the ferryl ion, a short-lived reactive oxygen species (ROS), coupling anode, cathode, and bulk Fenton chemistry in a single model validated against measured Fe­(II), dissolved oxygen (DO), hydrogen peroxide (H_2_O_2_), and the sum of Fe­(II) and Fe­(III) concentrations. Our findings predict the rapid turnover and low concentration (<1.80 μg/L) of the ferryl ion as a reactive intermediate species. Interestingly, the kinetic model predicts that lower electrocoagulation currents yield higher ferryl ion concentrations. Further, increased electrocoagulation time results in faster consumption of the ferryl ion after the electrocoagulation step. The implication is that these results provide a guiding framework for designing electrocoagulation processes.

## Graphical abstract

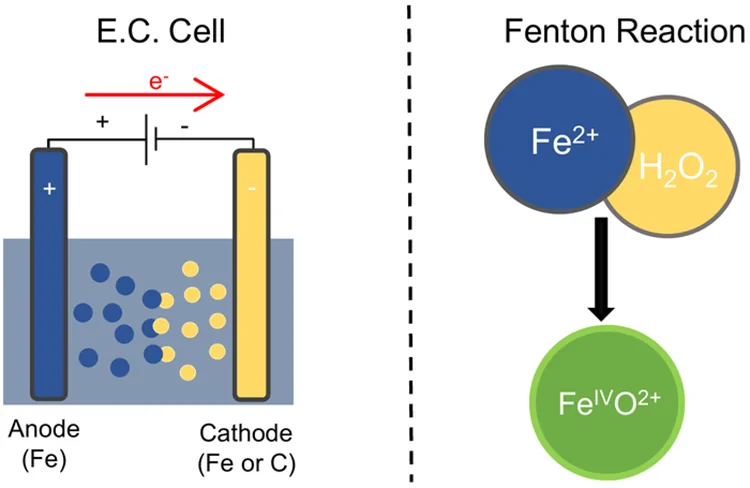

## 1 Introduction

Electrocoagulation has emerged as an innovative, versatile, and cost-effective method in the field of wastewater treatment, occupying a position in either primary or secondary treatment stages.^1–5^ The method has been proposed as an alternative to traditional coagulation-flocculation and chemical treatments due to its efficiency in chemical oxygen demand (COD) reduction and contaminant removal in municipal and industrial wastewater.^4–19^ In the electrocoagulation process, an electric current is applied to a sacrificial anode (usually iron or aluminum), which oxidizes to release metal ions into the wastewater. These metal ions then react with water to form metal hydroxides, which serve as in situ coagulants that react with and aggregate suspended solids, colloidal particles, dissolved organic pollutants, and other contaminants. Concurrently, gas bubbles from the hydrogen evolution reaction produced at the cathode aid in the flotation and removal of the flocs. In advanced electrocoagulation systems integrated with electro-Fenton mechanisms, the modified cathode (typically carbon-based) produces hydrogen peroxide (H_2_O_2_) via the two-electron reduction of oxygen. The generated species combine in the bulk solution to trigger the Fenton reaction, releasing highly reactive radicals that degrade or neutralize contaminants into nontoxic compounds.^20–23^ However, because of its complex reaction network, electrocoagulation remains difficult to model computationally.

Recent advancements in the kinetic modeling of wastewater treatment processes reflect an increasing effort to improve predictive accuracy, operational efficiency, and adaptability of models to real-world conditions.^10,24–26^ One key research focus is the influence of operating parameters related to the electrocoagulation process, including electrode type, current density, and pH control, because of their impact on floc formation, energy consumption, and process efficiency. Specifically, increasing the experimental current density in electrocoagulation enhances contaminant removal by accelerating anodic dissolution and increasing coagulant and bubble generation, thereby promoting floc formation and electrofloatation.^16,27,28^ However, excessively high current densities can reduce performance through increased energy consumption, parasitic side reactions, and electrode passivation.^29,30^ For reactive oxygen species (ROS)-driven inactivation specifically, higher current densities promote competing cathodic hydrogen evolution that reduces H_2_O_2_ faradaic efficiency, while elevated Fe­(II) concentrations can scavenge oxidants and shift production toward weaker species such as hydroperoxyl radicals (HO_2_^•^).^6,30^ Also, acidic pH favors rapid dissolution of sacrificial electrodes and the formation of soluble metal ions, which can improve coagulation kinetics for certain dissolved contaminants.^10,31^ Neutral pH in electrocoagulation typically achieves optimal performance due to the formation of amorphous metal hydroxide flocs (e.g., Al­(OH)_3_ or Fe­(OH)_3_) with high adsorption capacity.^32,33^

Despite these advancements, most electrocoagulation modeling efforts have remained predominantly focused on contaminant-specific systems, particularly phosphate removal in industrial effluents and COD reduction,^24–26^ while mechanistic models addressing reactive oxygen species, which are important for virus inactivation, remain limited. Kinetic models that omit the ferryl ion can describe iron dosing and the coagulative removal of contaminants, but they cannot mechanistically account for the in situ oxidation that distinguishes aerated iron electrocoagulation and peroxicoagulation from conventional coagulation. Including the ferryl ion explicitly links the oxidant to measurable species: Fe­(II), dissolved oxygen (DO), and H_2_O_2_, and allows the relative contributions of ferryl and competing oxidants to be examined quantitatively. Current models often overlook the coupling among interfacial electrochemistry, bulk hydrolysis, and reactive oxidant generation, leaving a gap in comprehensive kinetic models that account for the heterogeneous nature of municipal wastewater and the concentration dependencies of its chemical species. Addressing this gap will aid in understanding the electrocoagulation process and identifying paths to further innovate within the field.

Here, we develop and validate a kinetic model for an electrocoagulation process in moderate to slightly acidic media for water treatment. Guided by experimental data, this model provides insight into the time-dependent concentrations of target chemical species vital to the wastewater treatment process at varying pH values. The experimental system^34^ employed two cell configurations to which a constant current was applied: an iron anode–carbon cathode configuration (Fe–C) and an iron anode–iron cathode configuration (Fe–Fe) with synthetic wastewater at pH values of 5.0, 5.5, 6.0, and 6.5 (see Supporting Information Section S1). Relevant electrochemical surface reactions at each respective electrode were considered as well as homogeneous reactions (i.e., Fenton reaction involving the ferryl ion (Fe^IV^O^2+^)) occurring in the bulk electrolyte. Using experimental electrocoagulation data,^34^ the model herein implements nonlinear regression to estimate best-fit values for kinetic parameters. These parameters included exchange-current densities (*i*_o_) and overpotentials (η_s_) for anodic dissolution, water reduction, and peroxide formation at the cathode, the volumetric mass transfer coefficient for oxygen mass transfer (*k*_L_ *a*), and rate constants for the Fenton reaction and the reduction of the ferryl ion (Fe^IV^O^2+^). From this model, the time-dependent concentrations of Fe­(II), Fe­(III), dissolved oxygen (DO), hydrogen peroxide (H_2_O_2_), and the short-lived ferryl ion were predicted.

Our kinetic model predicts an increase in ferryl ion concentration with pH for both configurations, with predicted levels in the range of 10^–4^–10^–3^ mg/L. Previous experimental work^34^ showed higher virus control for iron anode–carbon cathode (Fe–C) electrocoagulation at pH 5.5 and 6.5 compared to an iron anode–iron cathode (Fe–Fe) configuration at the same pH, indirectly suggesting that our findings on ferryl ion concentration may support the prior observation of virus inactivation.

## 2 Methods

### 2.1 Batch Reactor Model

A batch reactor model assuming a perfectly well-mixed and spatially uniform liquid phase was used to describe the kinetics of the electrocoagulation process.^34^ Three electrochemical surface reactions are considered: one at the anode and two at the cathode, as well as two homogeneous reactions in the electrolyte (Figure 1).

**Figure 1:**
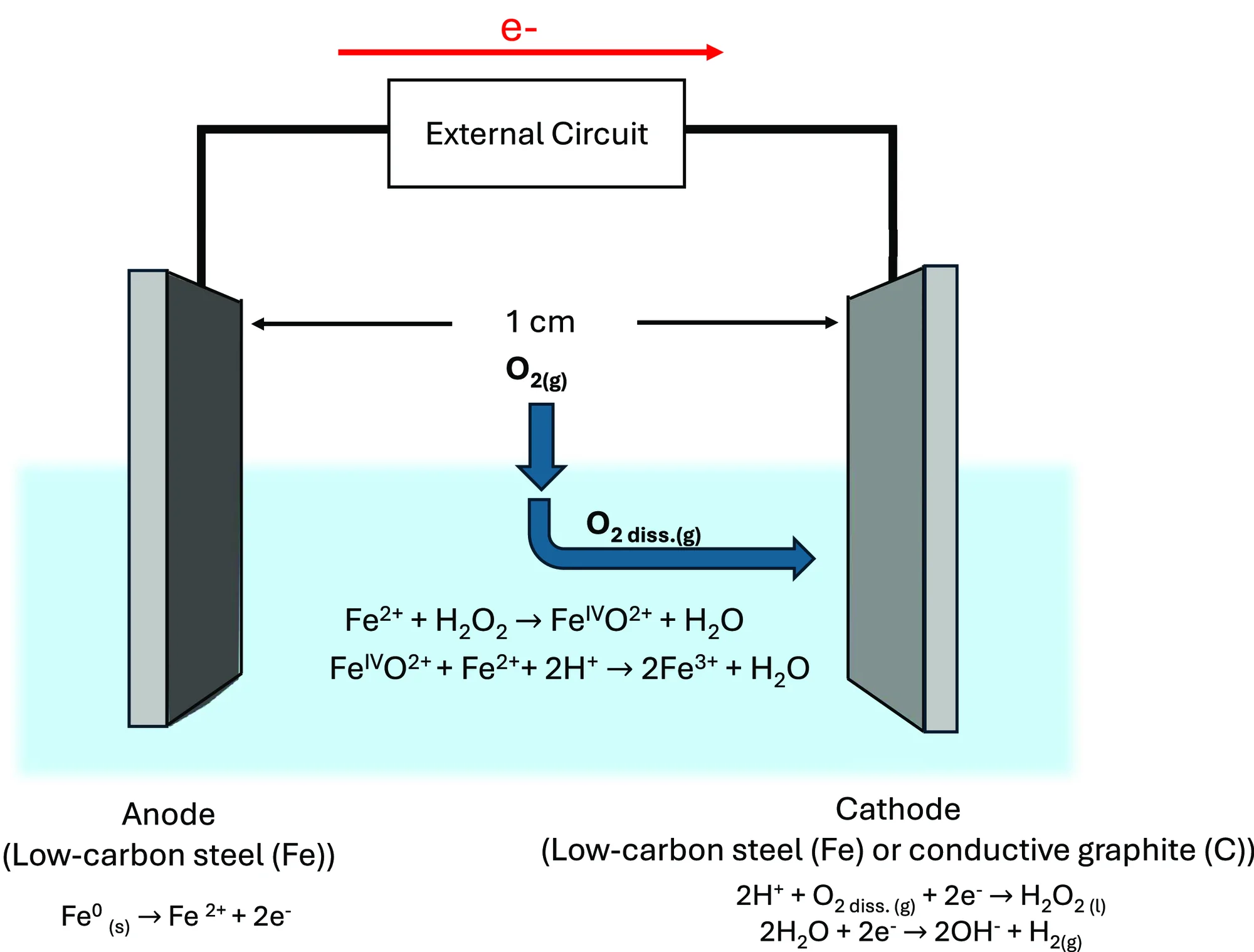
Schematic of electrocoagulation process.

At the iron anode, the oxidation of zero-valent iron was considered^4,5,10,35^

1 $$Fe^{0}\left(s\right)\to Fe^{2+}+2e^{-}$$

At the cathode, the two reactions considered were the peroxide formation reaction and hydrogen evolution reaction^4,10,36–39^

2 $$2H^{+}+O_{2\left(\mathrm{dissolved}\right)}+2e^{-}\to H_{2}O_{2\left(l\right)}$$

3 $${2H}_{2}O+2e^{-}\to 2OH^{-}+H_{2\left(g\right)}$$

In the bulk electrolyte, the homogeneous Fenton reaction 4 was modeled as Fe­(II) reacting with hydrogen peroxide to form Fe^IV^O^2+^ rather than hydroxyl radical (^•^OH) because of the studied pH range (5.0–6.5). Although the classical Fenton reaction typically forms ^•^OH under highly acidic conditions, mechanistic studies have shown a pH-dependent shift toward ferryl ion formation at higher pH levels.^40,41^ Koppenol^40^ explains that above pH 5, the Fenton reaction becomes “first order in HO^–^ and oxygen atom transfer has been detected, which indicates formation of oxidoiron (2^+^), Fe^IV^O^2+^”, and Bataineh et al.^41^ reported a mechanistic changeover from hydroxyl radical to Fe­(IV) near pH 6–7. As a result, the ferryl ion was selected as the more appropriate oxidant for the homogeneous Fenton reaction for the studied pH range

4 $$Fe^{2+}+H_{2}O_{2}\to F{e^{\mathrm{IV}}O}^{2+}+H_{2}O$$

In addition, the reaction between Fe^2+^, Fe^IV^O^2+^, and H^+^ to form Fe^3+^ and water was also considered. This reaction accounts for the subsequent consumption of the ferryl ion after its formation by the Fenton reaction^42,43^

5 $${{\mathrm{Fe}}^{\mathrm{IV}}O}^{2+}+Fe^{2+}+2H^{+}\to {2\mathrm{Fe}}^{3+}+H_{2}O$$

The electrochemical kinetics of the system was considered using Faraday’s law (eq 6), the relationship between charge and current (eq 7), the Butler–Volmer equation (eq 8), and the relationship between the assumed anodic transfer coefficients and the number of electrons transferred (eq 9).^44,45^

6 $$m_{j}=\frac{M_{j}Q}{\mathrm{n F}}$$

7 Q=It

8 $$i=i_{o}\left[\exp\left(\frac{\alpha_{a}F\eta_{s}}{\mathrm{RT}}\right)-\exp\left(-\frac{\alpha_{c}F\eta_{s}}{\mathrm{RT}}\right)\right]$$

9 $$\alpha_{a}+\alpha_{c}=n$$

To account for concentration dependencies in the Butler–Volmer equation, the following variation was used (eq 10)^45^

10 $$i=i_{o,\mathrm{ref}}\left[{\left(\frac{C_{\mathrm{ox}.}}{C_{\mathrm{ox}.,\mathrm{ref}}}\right)}^{\gamma_{1}}\exp\left(\frac{\alpha_{a}F\eta_{s}}{\mathrm{RT}}\right)-{\left(\frac{C_{r\mathrm{ed}.}}{C_{r\mathrm{ed}.,\mathrm{ref}}}\right)}^{\gamma_{2}}\exp\left(-\frac{\alpha_{c}F\eta_{s}}{\mathrm{RT}}\right)\right]$$

As a result, the expansions of the Butler–Volmer equation for each electrochemical reaction become

11 $$i_{1}=i_{o,1,\mathrm{ref}}\left((\frac{c_{{\mathrm{Fe}}^{0}}}{c_{{\mathrm{Fe}}^{0},\mathrm{ref}}})\exp\left(\frac{\alpha_{a}F\eta_{s}}{\mathrm{RT}}\right)-(\frac{c_{{\mathrm{Fe}}^{2+}}}{c_{{\mathrm{Fe}}^{2+},\mathrm{ref}}})\exp\left(\frac{-\alpha_{c}F\eta_{s}}{\mathrm{RT}}\right)\right)$$

$$i_{2}=i_{o,2,\mathrm{ref}}\left({\left(\frac{c_{H^{+}}}{c_{H_{\mathrm{ref}}^{+}}}\right)}^{2}\left(\frac{c_{O_{2}}}{c_{O_{2},\mathrm{ref}}}\right)\exp\left(\frac{\alpha_{a}F\eta_{s}}{\mathrm{RT}}\right)-\left(\frac{c_{H_{2}O_{2}}}{c_{H_{2}O_{2},\mathrm{ref}}}\right)\exp\left(\frac{-\alpha_{c}F\eta_{s}}{\mathrm{RT}}\right)\right)$$ 12

$${i_{3}=i}_{o,3,\mathrm{ref}}\left({\left(\frac{c_{H_{2}O}}{c_{H_{2}O,\mathrm{ref}}}\right)}^{2}\exp\left(\frac{\alpha_{a}F\eta_{s}}{\mathrm{RT}}\right)-{\left(\frac{c_{{\mathrm{OH}}^{-}}}{c_{{\mathrm{OH}}_{\mathrm{ref}}^{-}}}\right)}^{2}\left(\frac{c_{H_{2}}}{c_{H_{2,\mathrm{ref}}}}\right)\exp\left(\frac{-\alpha_{c}F\eta_{s}}{\mathrm{RT}}\right)\right)$$ 13

where γ_1_ and γ_2_ were replaced by the stoichiometric coefficients of the associated species in each reaction.

To determine the molar flux of each species in the system, eq 6 was modified to provide a molar flux (*N_j_*) in units mol/m^2^s

14 $$m\mathrm{olar flux}\;\left(N_{j}\right)=\frac{i}{\mathrm{nF}}$$

where *i* is the net current density from eq 10, *n* is the number of electrons transferred per equivalent of the electrochemical reaction, and *F* is Faraday’s constant. By combining eqs 10 and 14, the following equation was used to determine the molar flux of each species (*N_j_*)­

15 $$N_{1}=\frac{i_{o,1,\mathrm{ref}}}{\mathrm{nF}}\left((\frac{c_{{\mathrm{Fe}}^{0}}}{c_{{\mathrm{Fe}}^{0},\mathrm{ref}}})\exp\left(\frac{\alpha_{a}F\eta_{s}}{\mathrm{RT}}\right)-(\frac{c_{{\mathrm{Fe}}^{2+}}}{c_{{\mathrm{Fe}}^{2+},\mathrm{ref}}})\exp\left(\frac{-\alpha_{c}F\eta_{s}}{\mathrm{RT}}\right)\right)$$

$$N_{2}=\frac{i_{o,2,\mathrm{ref}}}{\mathrm{nF}}\left({\left(\frac{c_{H^{+}}}{c_{H_{\mathrm{ref}}^{+}}}\right)}^{2}\left(\frac{c_{O_{2}}}{c_{O_{2},\mathrm{ref}}}\right)\exp\left(\frac{\alpha_{a}F\eta_{s}}{\mathrm{RT}}\right)-\left(\frac{c_{H_{2}O_{2}}}{c_{H_{2}O_{2},\mathrm{ref}}}\right)\exp\left(\frac{-\alpha_{c}F\eta_{s}}{\mathrm{RT}}\right)\right)$$ 16

17 $$N_{3}=\frac{i_{o,3,\mathrm{ref}}}{\mathrm{nF}}\left({\left(\frac{c_{H_{2}O}}{c_{H_{2}O,\mathrm{ref}}}\right)}^{2}\exp\left(\frac{\alpha_{a}F\eta_{s}}{\mathrm{RT}}\right)-{\left(\frac{c_{{\mathrm{OH}}^{-}}}{c_{{\mathrm{OH}}_{\mathrm{ref}}^{-}}}\right)}^{2}\left(\frac{c_{H_{2}}}{c_{H_{2,\mathrm{ref}}}}\right)\exp\left(\frac{-\alpha_{c}F\eta_{s}}{\mathrm{RT}}\right)\right)$$

For reaction 2, the Tafel approximation was implemented under the assumption that the electrochemical surface reactions were irreversible. Therefore, eq 16 reduces to

18 $$i_{2}=\frac{i_{o,2\;\mathrm{ref}}}{\mathrm{nF}}\left({\left(\frac{c_{H^{+}}}{c_{H_{\mathrm{ref}}^{+}}}\right)}^{2}\left(\frac{c_{O2}}{c_{O2,\mathrm{ref}}}\right)\exp\left(\frac{\alpha_{a}F\eta_{s}}{\mathrm{RT}}\right)\right)$$

The molar fluxes were multiplied by a scaling factor determined by the division of the submerged electrode surface (m^2^) by the total volume of the electrolyte (m^3^)­

19 $$s\mathrm{caling factor}\;\left(â\right)=\frac{s\mathrm{ubmerged electrode surface area}}{t\mathrm{otal liquid volume}}$$

The scaling factor, when multiplied by the molar fluxes, provided the rate equations for the electrochemical reactions

20 $$\mathrm{reaction rate}\;\left(r_{j}\right)\;=\left(\mathrm{molar flux}\right)\;\left(â\right)$$

Considering the areal current of the experiment, the net current density (*i*), at both the anode and cathode is 9.5 A/m^2^. As a result, eqs 11–13 were considered as

21 $$i_{1}=9.5\frac{A}{m^{2}}$$

22 $$i_{2}+i_{3}=9.5\frac{A}{m^{2}}$$

Therefore, the reaction rates (*r_j_*, units: mol/m^3^ s) of the electrochemical reactions can be expressed as

23 $$r_{1}=\left(\frac{9.5}{\mathrm{nF}}\right)\;\left(â\right),\mathrm{reaction}\;1:{\mathrm{Fe}}^{0}\left(s\right)\to {\mathrm{Fe}}^{2+}+2e^{-}$$

24 $$r_{2}=\left(\frac{i_{2}}{\mathrm{nF}}\right)\;\left(â\right),\mathrm{reaction}\;2:2H^{+}+O_{2\left(\mathrm{dissolved}\right)}+2e^{-}\to H_{2}O_{2\left(1\right)}$$

25 $$r_{3}=\left(\frac{{9.5-i}_{2}}{\mathrm{nF}}\right)\;\left(â\right),\mathrm{reaction}\;3:2H_{2}O+2e^{-}\to 2{\mathrm{OH}}^{-}+H_{2\left(g\right)}$$

The reaction rate for the Fenton reaction is represented as

26 $$r_{4}=k_{4}\left(C_{{\mathrm{Fe}}^{2+}}\right)\left(C_{H_{2}O_{2}}\right)$$

The ferryl ion is consumed by its reaction with ferrous iron, which in acidic media proceeds as shown in eq 5 in the main text. Taking this step to be elementary, the corresponding rate of ferryl ion consumption is shown in eq 27.

27 $$r_{5}={k^{\prime }}_{5}\left(C_{{\mathrm{FeO}}^{2+}}\right)\left(C_{{\mathrm{Fe}}^{2+}}\right){\left(C_{H^{+}}\right)}^{2}$$

where *k*_5_′ is the intrinsic rate constant and *C*_H^+^_ is the proton concentration. Because the pH variation is minimal over the reactive period of the given experiment, the proton concentration is treated as constant. For simplicity, (*C*_H^+^_)^2^ is therefore absorbed into an effective rate constant *k*_5_, and the ferryl-consumption rate used in the model is represented as

27b $$r_{5}=k_{5}\left(C_{{\mathrm{FeO}}^{2+}}\right)\left(C_{{\mathrm{Fe}}^{2+}}\right)\mathrm{with}\;K_{5}=K_{5}^{\prime }{\left(C_{H^{+}}\right)}^{2}$$

Oxygen mass transfer was also observed during the experimental procedure. To account for this, the following equation was included

28 $$r_{6}=k_{L}a\left(C_{O_{2}}^{\mathrm{eq}.}-C_{O_{2}}\right)$$

The mass balance equations for each species concentration were thus

29 $$\frac{d}{dt}C_{{\mathrm{Fe}}^{2+}}=r_{1}-r_{4}-r_{5}$$

30 $$\frac{d}{dt}C_{H^{+}}=0$$

31 $$\frac{d}{dt}C_{O_{2}}=-r_{2}+r_{6}$$

32 $$\frac{d}{dt}C_{H_{2}O_{2}}=r_{2}-r_{4}$$

33 $$\frac{d}{dt}C_{{\mathrm{Fe}}^{3+}}=r_{4}+r_{5}$$

34 $$\frac{d}{dt}C_{OH^{-}}=0$$

35 $$\frac{d}{dt}\left[s\mathrm{um of}{\;\mathrm{Fe}}^{2+}\;\&\;{\mathrm{Fe}}^{3+}\right]=\frac{dC_{{\mathrm{Fe}}^{2+}}}{dt}+\frac{dC_{{\mathrm{Fe}}^{3+}}}{dt}$$

36 $$\frac{d}{dt}C_{{{\mathrm{Fe}}^{\mathrm{IV}}O}^{2+}}=r_{4}-r_{5}$$

Equation 31 represents a fixed-pH approximation in which H^+^ concentrations are prescribed according to the target pH condition and are not treated as dynamic state variables. This simplification was adopted to isolate the effects of pH on iron redox chemistry, oxygen reduction, hydrogen peroxide formation, and ferryl ion generation. The experimental pH varied by less than 0.2 pH units during the 60 min experiments due to the nature of the feedwater (alkalinity of 100 mg/L as CaCO_3_). Therefore, we assumed the pH and the concentrations of protons (H^+^) and hydroxyl ions (OH^–^) of the bulk solution to be relatively constant. Electrode corrosion and iron dissolution were negligible in control experiments, showing only minimal iron dissolution (0.2–0.4 mg/L) across the investigated pH range. Anode passivation was also insignificant because iron concentrations closely followed Faraday’s law and because electrocoagulation was performed for only 60 min. Hence, the model is considered strictly applicable only for short-term electrocoagulation in the absence of significant corrosion or passivation.

### 2.2 Determination of Unknown Parameters

In MATLAB, Ode23s was used to numerically integrate eqs 23–37 via the modified Rosenbrock method.^46^ Initial conditions were taken from bulk initial species concentrations as shown in Table 1. The five unknown parameters are *i*_o,2_, η_s,2_, *k*_4_, *k*_5_, and *k*_L_ *a*, which are sensitive to system-specific factors such as electrode material, surface roughness, stir rate, and system geometry. To calculate the error associated with the given values for the five unknown parameters, the predicted concentration values from our model for the target species (Fe­(II), sum of Fe­(II) & Fe­(III), dissolved oxygen and H_2_O_2_) were compared with experiment using the equation below

$$\sigma =\sqrt{\frac{\sum_{j}^{M}\sum_{i}^{N}{\left(\frac{C_{j,i}^{\exp}-C_{j,i}^{calc}}{C_{j,i}^{\exp}}\right)}^{2}}{M\left(N-1\right)}}$$

**Table 1:** Physical Constants and Initial Conditions Utilized for the Kinetic Model

parameter	value	unit	source
*n*	2	equivalent/mol	
*F*	96,485	C/equivalent	
*R*	8.314	J/mol K	
*T*	298	K	
*C*_O_2__^eq.^	2.70 × 10^–1^	mol/m^3^	This work
*C*_Fe^2+^(*t*=o)_	1.00 × 10^–3^	mol/m^3^	This work
*C*_H_2_O_2_(*t*=o)_	1.00 × 10^–5^	mol/m^3^	This work
*C*_Fe^3+^(*t*=o)_	1.00 × 10^–5^	mol/m^3^	This work
*C*_total Fe(*t*=o)_	1.01 × 10^–3^	mol/m^3^	This work

where *M* is the number of species at *N* reaction times.

For each experimental data set, an initial guess for the five unknown parameters was made on the basis of literature values. Nonlinear least-squares regression was then performed using the MATLAB fminsearch package to find a minimum in the five-dimensional parameter space. The resulting set of parameter values was then perturbed by a random factor between 0 and 1 (using the MATLAB rand function) and used as the initial guess for a subsequent minimization to check for local vs global minima. This cycle was repeated a total of three times such that for each experiment there were four sets of parameter values fit to the data. Those values were then averaged, and the standard deviation was calculated. All reactions and equations used in the modeling approach were the same for both the iron anode–carbon cathode and iron anode–iron cathode configurations.

## 3 Results and Discussion

### 3.1 Fitting of the Bath Reactor Model to the Experimental Data

The batch reactor model was fit to the experimental data,^34^ resulting in estimates of unknown kinetic parameters outlined in Tables 2 and 3. Figures 2 and 3 show the concentrations of the experimentally monitored species, along with the fitted model for electrocoagulation at pH levels of 5.0, 5.5, 6.0, and 6.5 in the iron anode–carbon cathode and iron anode–iron cathode cells, respectively. In both configurations, the best-fit version of the model reasonably predicted the concentration profiles of Fe­(II), dissolved oxygen, H_2_O_2_, and the “sum of Fe­(II) and Fe­(III)” for all pH levels over the 60 min period. Fe­(II) and H_2_O_2_ concentrations increased during electrolysis due to their formation as per eqs 1–3. The sum of Fe­(II) and Fe­(III) concentration also increased during electrolysis primarily because of the continuous oxidation of zero-valent iron to ferrous ions. Post-electrolysis, Fe­(II) and H_2_O_2_ consumption occurred due to the Fenton reaction (eq 4), which formed Fe^IV^O^2+^.^40,41^ Dissolved oxygen was consumed during electrolysis due to the hydrogen peroxide (H_2_O_2_) generation reaction (eq 2). After electrolysis, dissolved oxygen concentrations increased due to oxygen mass transfer between the air and the electrolyte (eq 29). Both cell configurations followed similar trends in concentration profiles with time.

**Figure 2:**
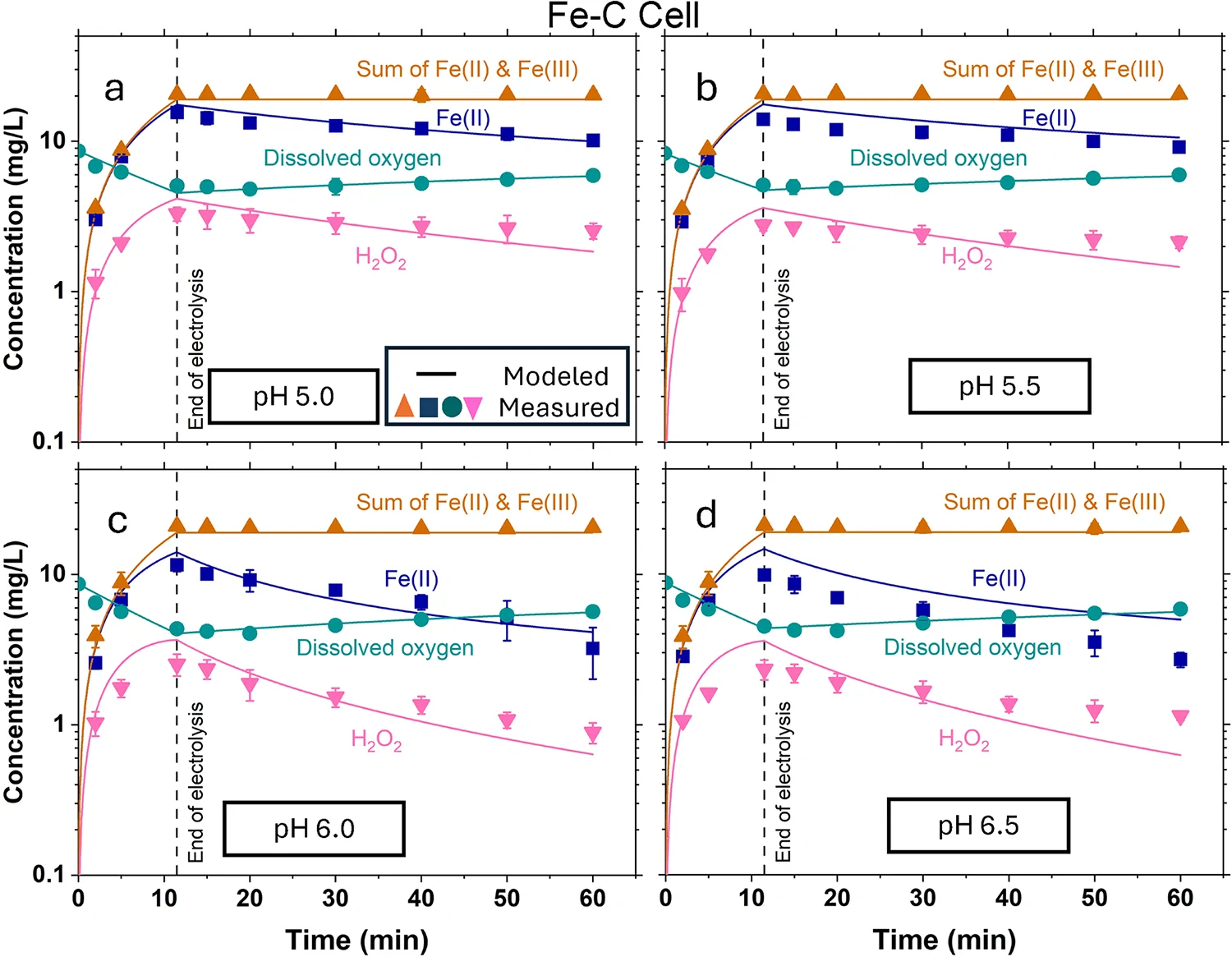
Comparison of experimental and modeled concentrations during and after electrolysis: (a) pH 5.0, (b) pH 5.5, (c) pH 6.0, and (d) pH 6.5 in an Fe–C cell, where iron was the anode and carbon was the cathode. The applied current was 9.5 A/m^2^ for the first 11.5 min. Experimental data are shown as points, and the model is represented as a solid line.

**Figure 3:**
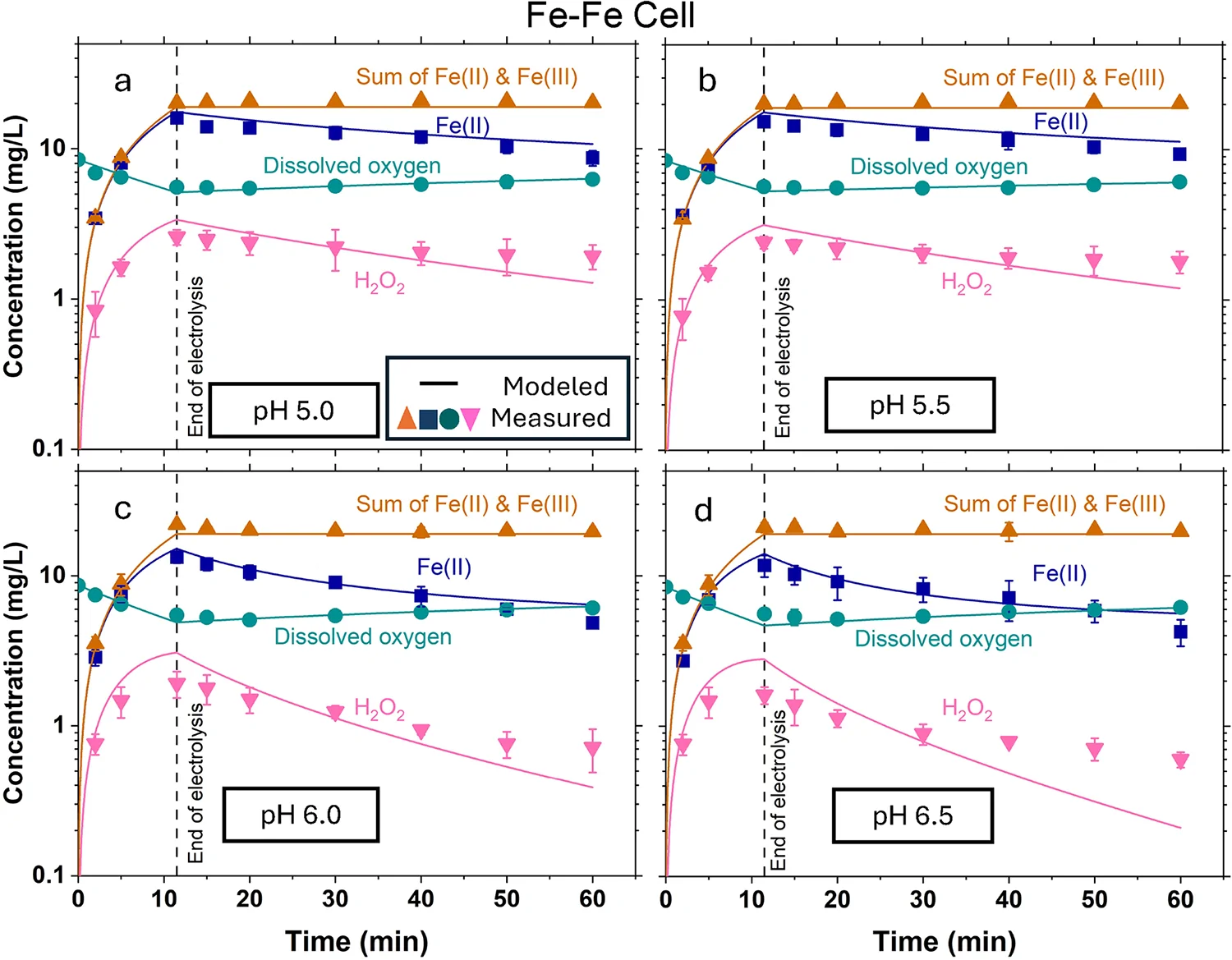
Comparison of experimental and modeled concentrations during and after electrolysis: (a) pH 5.0, (b) pH 5.5, (c) pH 6.0, and (d) pH 6.5 in an Fe–Fe cell. Experimental data are shown as data points, and the model is represented as a solid line.

**Table 2:** Model-Estimated Parameter Values for the Iron Anode–Carbon Cathode (Fe–C) Cell at Different pH Values

pH	*i*_o,2_ (A/m^2^)	η_s,2_ (V)	*k*_4_ (m^3^/mol s)	*k*_5_ (m^3^/mol s)	*k*_L_ *a* (s^–1^)	error (%)
5.0	1.41 × 10^–6^	2.68 × 10^–1^	1.21 × 10^–3^	180	1.36 × 10^–4^	5.31
5.5	9.64 × 10^–7^	2.68 × 10^–1^	1.28 × 10^–3^	160	1.14 × 10^–4^	5.96
6.0	3.53 × 10^–7^	3.11 × 10^–1^	4.92 × 10^–3^	156	1.42 × 10^–4^	8.47
6.5	1.31 × 10^–6^	2.75 × 10^–1^	4.32 × 10^–3^	116	1.16 × 10^–4^	9.52

**Table 3:** Model-Estimated Parameter for the Iron Anode–Iron Cathode (Fe–Fe) Cell at Different pH Values

pH	*i*_o,2_ (A/m^2^)	η_s,2_ (V)	*k*_4_ (m^3^/mol s)	*k*_5_ (m^3^/mol s)	*k*_L_ *a* (s^–1^)	error (%)
5.0	9.58 × 10^–7^	2.66 × 10^–1^	1.38 × 10^–3^	264	1.51 × 10^–4^	6.12
5.5	1.01 × 10^–6^	2.28 × 10^–1^	1.38 × 10^–3^	85.9	9.96 × 10^–5^	6.34
6.0	9.55 × 10^–7^	2.70 × 10^–1^	4.44 × 10^–3^	106	1.55 × 10^–4^	9.11
6.5	1.88 × 10^–6^	2.56 × 10^–1^	4.97 × 10^–3^	21.2	1.69 × 10^–4^	11.88

### 3.2 Comparing the Best-Fit Kinetic Parameters with pH and Cell Configuration

Figure 4 shows model estimates for the five unknown parameters across all four pH levels and two configurations. As shown in Figure 4a, the exchange-current density for reaction 2, *i*_o,2_, provides insight into the rate of reaction for the electrochemical formation of hydrogen peroxide at the cathode. For both configurations, *i*_o,2_ is similar for pH values of 5.0 and 5.5. At the slightly higher pH values of 6.0 and 6.5, *i*_o,2_ was larger for the iron anode–iron cathode configuration. This result points to faster reaction kinetics for peroxide formation on the iron cathode as compared with the carbon one.

**Figure 4:**
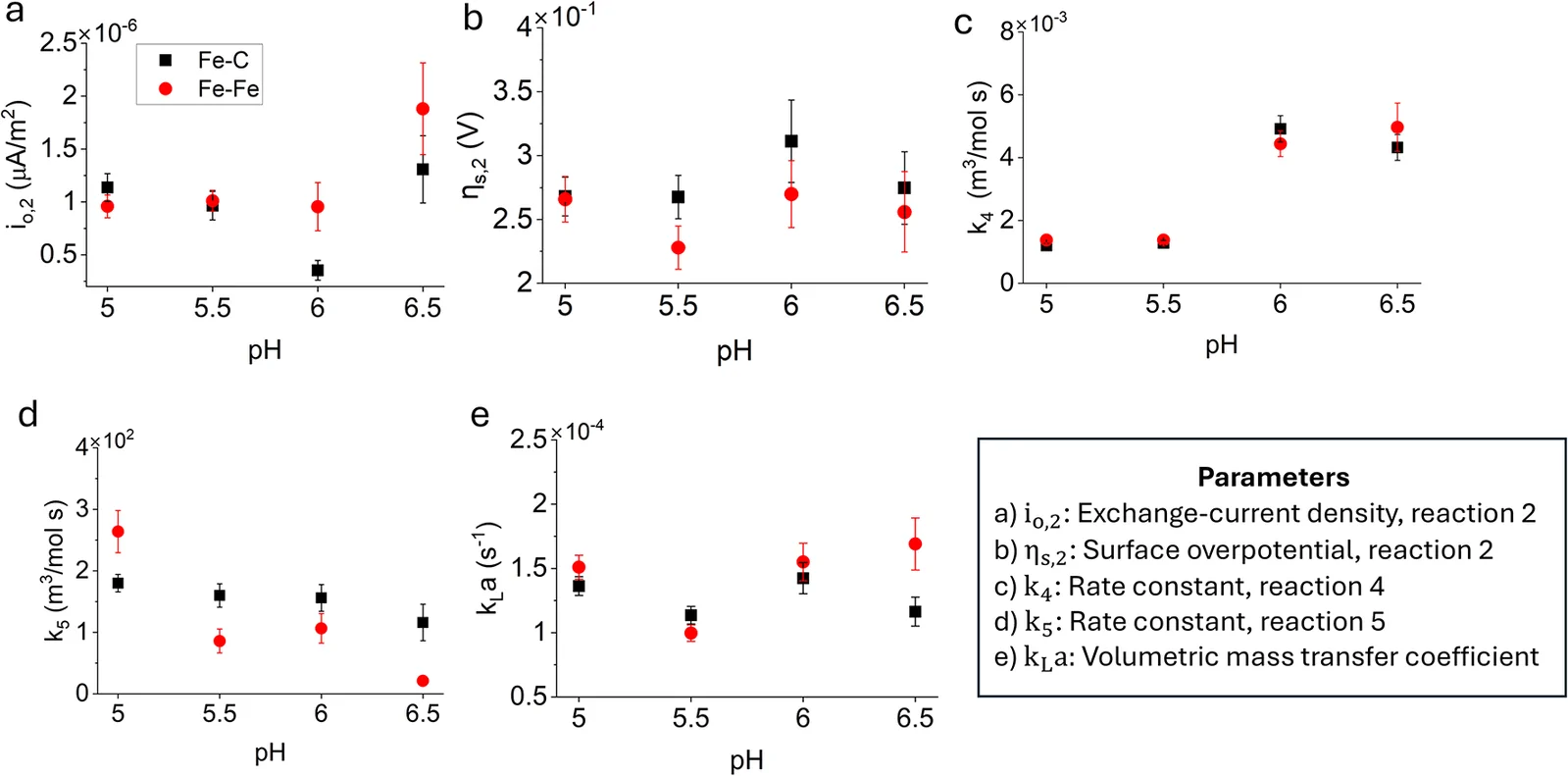
Comparison of best-fit kinetic parameters (a) i_o,2_: Exchange-current density for reaction 2; (b) η_s,2_: Surface overpotential for reaction 2; (c) k_4_: Rate constant for reaction 4; (d) k_5_: Rate constant for reaction 5; and (e) k_L_a: Volumetric mass transfer coefficient between iron anode–carbon cathode (Fe–C) and iron anode–iron cathode (Fe–Fe) configurations with varying electrocoagulation pH values.

Figure 4b shows the surface overpotential, η_s,2_, for the cathodic formation of H_2_O_2_ in reaction 2. This parameter affects the production of H_2_O_2_ as shown in the Tafel approximation in eq 18. The surface overpotential is an indication of the amount of additional driving force required for the electrochemical reaction. The positive values for the best-fit surface overpotential are consistent with the cathodic formation of peroxide. In this case, the overpotential values are quite close (with iron anode–carbon cathode being marginally higher) when both configurations are compared across all four pH levels.

Figure 4c,d represents the best-fit estimates for parameters related to the production and consumption of the ferryl ion (Fe^IV^O^2+^). This is an important reactive species in electrocoagulation water treatment processes, but the ferryl ion is a highly reactive species that is difficult to directly observe.^40,41,43,47,48^ Therefore, there is scarce literature available for rate constants related to the formation of the ferryl ion via the Fenton reaction. In Koppenol’s^40^ work, the authors provide a rate constant for the Fenton reaction near neutral pH values by extrapolating rate constant values collected from previous research papers under both acidic and basic conditions. Our model estimates that the rate constant attributed to the Fenton reaction, *k*_4_, increases significantly at pH 6.0, which implies that near-neutral pH conditions favor the formation of the ferryl ion. The model estimates that *k*_4_ ranges from 1.21 × 10^–3^ at pH 5.0 and up to 4.97 × 10^–3^ m^3^/mol s at pH 6.5. This value is much smaller than the previously estimated value for the Fenton reaction at the same pH (1.59 m^3^/mol s).^40^ The lower rate constants from our model could be due to the limited number of reactions considered. However, our model estimates agree with literature reports of increased ferryl ion formation as pH increases above pH 5.^40,41,49,50^

As the ferryl ion is a short-lived, low-concentration species, one must also consider its consumption via the reaction in eq 5, for which Figure 4d shows the affiliated kinetic rate constant. Overall, the values are on the same order of magnitude as reported by Deng et al.^42^ (140 m^3^/mol s). Because *k*_5_ is much greater than *k*_4_, the consumption of the ferryl ion is significantly rapid, thus making its presence extremely short-lived. The value of *k*_5_ remained constant (within error) for the iron anode–carbon cathode configuration at all pH values studied. On the other hand, the iron anode–iron cathode cell’s value of *k*_5_ was highest at pH 5.0 (264 vs 180 m^3^/mol s for Fe–C) and then dropped below that of iron anode–carbon cathode values at higher pH values. This trend implies less consumption of the ferryl ion under the iron anode–iron cathode configuration.

The volumetric gas–liquid mass transfer coefficient for oxygen (*k*_L_ *a*) best-fit estimates are shown in Figure 4e. Overall, these values are similar for both configurations at pH 5.0, 5.5, and 6.0, as expected. For the iron anode–carbon cathode configuration, the model estimates are within 20% of each other with no clear trend. Literature values of *k*_L_ *a* are reported to be between 3.79 × 10^–3^ and 6.67 × 10^–3^ at 25 °C in water, depending on the method of measurement and experimental condition.^51,52^ The model’s best-fit values for *k*_L_ *a* ranged from 9.96 × 10^–5^ to 1.69 × 10^–4^ s^–1^. This difference is attributed to contrasts in our experimental configuration (geometry, stirring, etc.) versus those from the literature.

### 3.3 Prediction of the Ferryl Ion Concentration

Using the best-fit parameters from Tables 2 and 3, the evolution of the ferryl ion was predicted for the duration of the electrocoagulation process. Figure 5 shows the predicted ferryl ion concentration profiles for both configurations at varying pH values. Generally, the ferryl ion concentration initially increases in the electrolysis period, after which the concentration declines for the remaining time. This trend is understandable given the fact that the production and consumption of the ferryl ion are directly correlated to the production and subsequent consumption of Fe­(II) and H_2_O_2_, as shown in eqs 4 and 5. At pH values of 6.0 and 6.5 for both configurations, the ferryl ion concentrations were significantly higher than those at lower pH values. At pH 6.5, the ferryl ion concentrations were 5 × 10^–4^ and 1.8 × 10^–3^ mg/L for the iron anode–carbon cathode and iron anode–iron cathode cells, respectively. In contrast, at all other pH values, the ferryl ion concentrations were higher for the iron anode–carbon cathode configuration.

**Figure 5:**
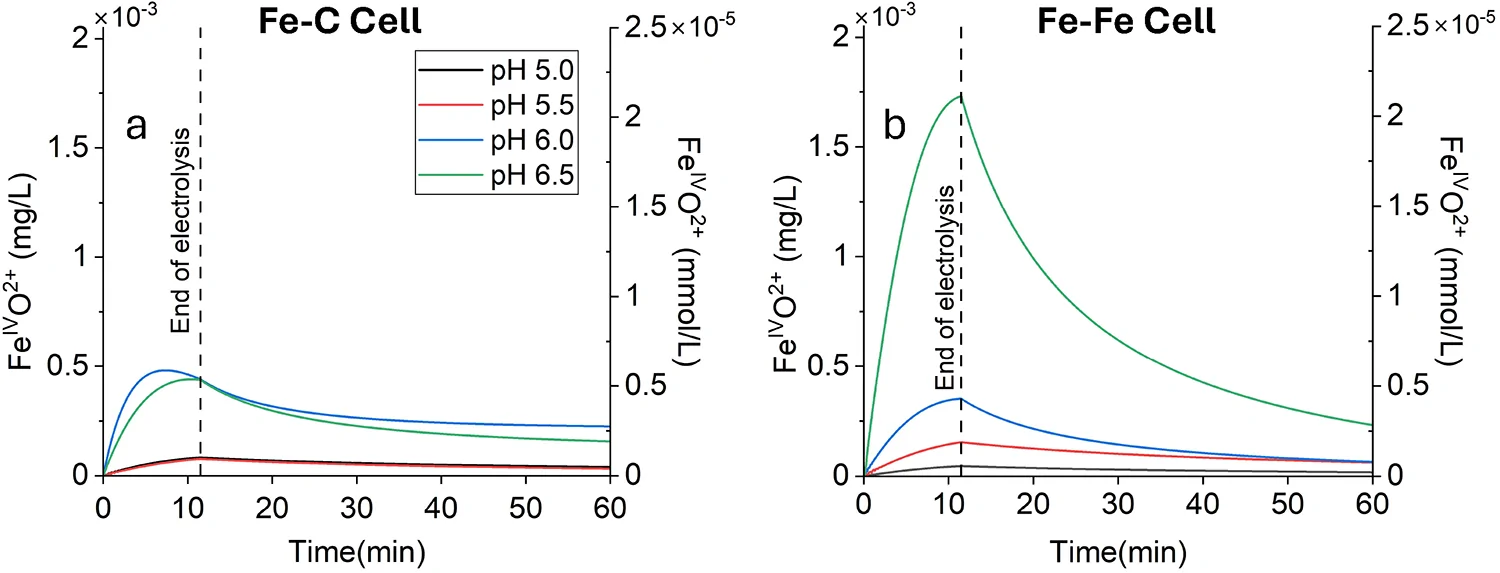
Fe^IV^O^2+^ concentration during electrocoagulation for the two cell configurations at different electrocoagulation pH values. (a) Iron anode–carbon cathode (Fe–C) and (b) iron anode–iron cathode (Fe–Fe) configurations.

Our kinetic model predicts low steady-state concentrations of ferryl ion during electrocoagulation, a result that is consistent with the known behavior of high-valency iron oxidants. As a high-valency reactive oxygen species (ROS), the ferryl ion is characterized by fast reaction kinetics and very short lifetimes.^40,41,50,53^ Consequently, the ferryl ion’s rapid formation is balanced by equally rapid consumption, yielding low steady-state concentrations, which the model predicts. Previous experimental studies have shown that iron electrocoagulation generates reactive oxygen species through electro-Fenton and Fenton-like pathways, including pathways leading to Fe­(IV) formation, and that these pathways contribute directly to log virus reduction values (LRV) beyond physical removal mechanisms.^34,54,55^ Therefore, the low ferryl concentrations predicted in Figure 5 reflect the model’s ability to represent the rapid turnover of a highly reactive oxidant whose localized generation, rather than bulk concentration, drives virus inactivation in electrocoagulation systems.

Across the pH range and configurations tested, our team’s prior experimental results^34^ demonstrated condition-dependent differences in virus inactivation. The prior experiments determined that the iron anode–carbon cathode pairing yielded more effective virus control, achieving virus inactivation below detection limits (LRVs ≥ 6.7) at pH 5.5 and 6.5 when compared to the iron anode–iron cathode pairing. This difference was attributed to the higher faradaic efficiency of H_2_O_2_ formation at the graphite cathode when compared to an iron cathode. As discussed earlier, H_2_O_2_ is an important precursor for ROS formation, including the ferryl ion. A comparison of these prior experimental results with the current model further informs the interpretation. For example, at pH 5.5, our model predicts H_2_O_2_ concentrations of 3.61 and 3.16 mg/L for the iron anode–carbon cathode and iron anode–iron cathode configurations at the conclusion of their respective electrolysis periods. Despite this higher H_2_O_2_ availability, the iron anode–carbon cathode configuration yields a lower predicted ferryl ion concentration (7.68 × 10^–5^ mg/L) compared to the iron anode–iron cathode configuration (1.55 × 10^–4^ mg/L). Since ferryl is formed directly from Fe­(II) and H_2_O_2_ through the Fenton reaction, its instantaneous concentration is governed by the available steady-state Fe­(II) rather than H_2_O_2_ alone. In the iron anode–carbon cathode configuration, the greater H_2_O_2_ availability accelerates Fe­(II) consumption, lowering the steady-state Fe­(II) concentration. This is consistent with previous experimental observations,^34^ where slightly lower Fe­(II) concentrations in the iron anode–carbon cathode configuration were reported, and this was attributed to faster Fe­(II) consumption driven by higher H_2_O_2_ availability. The higher H_2_O_2_ concentration at the end of the electrolysis period in the iron anode–carbon cathode cell continuously drives the Fenton reaction at a greater rate, generating more cumulative ROS exposure over the treatment period. This sustained oxidant production, rather than the instantaneous concentration of any single ROS species, provides a more likely explanation for the higher virus inactivation observed experimentally in the iron anode–carbon cathode configuration.^34,56,57^

Taken together, the higher H_2_O_2_ production predicted for the iron anode–carbon cathode configuration and the mechanistic interpretation that this enables sustained Fenton chemistry, resulting in greater cumulative exposure to reactive oxygen species, support the conclusion that this configuration is more advantageous for virus removal at slightly acidic to near-neutral pH. We note that the model has challenges in that it does not specifically include additional factors faced in real experiments, such as electrode roughness and contributions from parallel radical or adsorption-based pathways, including hydroxide formation.^27,55,58,59^ Overall, the model adequately predicts the concentrations of major species, indicating its utility within the current framework.

### 3.4 Effects of Varying the Applied Current Density

Having validated the model, we next used the best-fit parameters from Tables 2 and 3 to predict the effects of current density (10–30 A/m^2^) on the concentrations of Fe­(II), sum of Fe­(II) and Fe­(III), H_2_O_2_, dissolved oxygen, and ferryl ion for the iron anode–carbon cathode cell at pH 6.5 (the configuration and pH that experimentally provided the best log virus reduction).^34^ Electrolysis time was adjusted to achieve target concentrations of 20 mg/L or 0.358 mol/m^3^ of Fe­(II) + Fe­(III). As shown in Figure 6a,d, a shorter electrolysis time is required to reach the target iron concentration as current density increases. As a result, the electrolysis time required decreases from 691 s at 10 A/m^2^ to 348 s at 20 A/m^2^ and then 231 s at 30 A/m^2^. As a result, the model estimates higher formation rates for Fe­(II) with increasing current density but also higher amounts of Fe­(II) remaining at the conclusion of the 60 min time period. This indicates a decreased consumption of Fe­(II) with increasing current density. As for the hydrogen peroxide concentration, Figure 6b shows that increasing the current density decreases the maximum amount of peroxide formed. This correlates with the decrease in electrolysis time, as peroxide formation occurs only at the cathode during electrolysis. With regard to dissolved oxygen, Figure 6c shows that increasing the current density leads to higher concentrations; this result stems from the lower consumption of oxygen at the cathode due to the shorter electrolysis time. Lastly, Figure 6e shows that the ferryl ion concentration decreases with increasing current density, because ferryl ion production is linked to hydrogen peroxide formation. The apparent inverse relationship arises from the termination criterion. Rather than running each simulation for a fixed time, electrolysis is terminated when the total dissolved iron (sum of Fe­(II) and Fe­(III)) reaches its target 20 mg/L. As a result, higher applied current densities reach this target in a shorter electrolysis time; for example, the time to reach 20 mg/L fell from 11.5 to 5 min to about 3.8 min. The ferryl ion is produced through the bulk-phase Fenton reaction, which depends on the accumulation of cathodically generated H_2_O_2_, which is a comparatively slow process. At higher applied current densities, the shortened electrolysis window allows less time for H_2_O_2_ to accumulate and react with Fe­(II), so the predicted ferryl concentration is correspondingly lower, despite the higher instantaneous reaction rates. In other words, the lower ferryl concentration reflects the reduced duration of the run, not suppression of ferryl formation by the current density.

**Figure 6:**
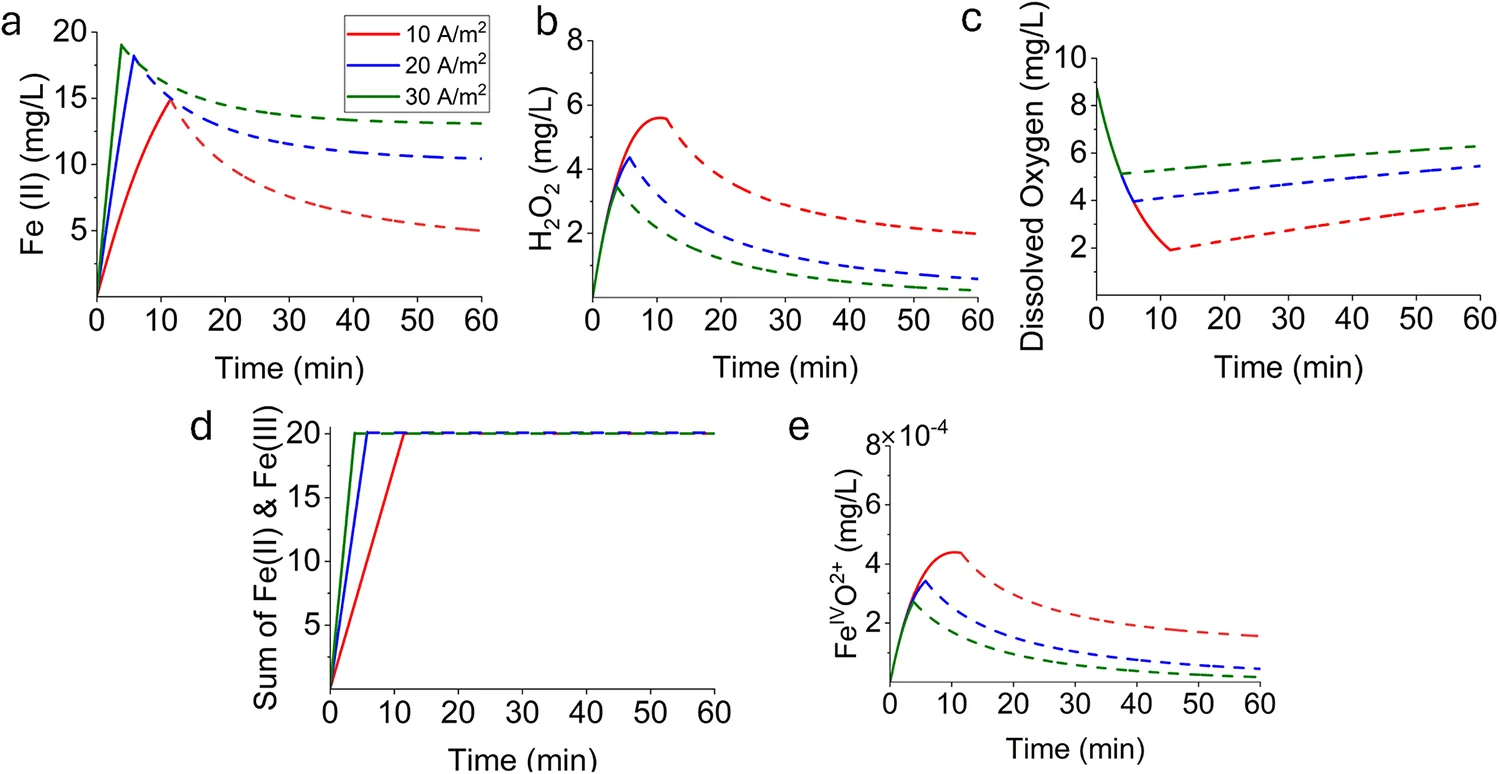
Concentration profiles for different applied current densities for electrocoagulation at pH 6.5 for the Fe–C cell. Concentrations of (a) ferrous iron (Fe­(II)), (b) hydrogen peroxide (H_2_O_2_), (c) dissolved oxygen, (d) sum of Fe­(II) and Fe­(III), (e) ferryl ion (Fe^IV^O^2+^) at pH 6.5 for current densities of 10, 20, and 30 A/m^2^. The solid lines represent the electrolysis period, while the dotted lines represent the time after electrolysis.

Analogous trends were observed across the remaining configurations and pH conditions with the iron anode–carbon cathode configuration at pH 5.0, 5.5, and 6.0 (Figures S1–S3) and the iron anode–iron cathode configuration at pH 5.0, 5.5, 6.0, and 6.5 (Figures S4–S7). Consequently, we expect that increased current densities (for equivalent charge passed) would result in visibly lower rates of pollutant removal or virus inactivation from wastewater because of the lower rates of peroxide and ferryl ion production.

### 3.5 Effects of Varying the Electrolysis Time

As shown in Figure 7, the model was used to predict how electrolysis time influences the evolution of each species for the Fe–C configuration at pH 6.5, at a fixed current density of 10 A/m^2^, under half, current, and double the experimental electrolysis time. Because the applied current is held constant, the rate of anodic iron dissolution is identical in all three cases, so total dissolved iron accumulates along a common trajectory and plateaus at the moment electrolysis ceases, fixing the final iron dose at approximately 10, 20, and 40 mg/L in direct proportion to the treatment time (Figure 7d). This additional iron propagates through the system: Fe­(II) exhibits a higher maximum at each cutoff (Figure 7a), while dissolved oxygen is consumed progressively to a lower minimum, reaching about 0.7 mg/L for the case of twice the electrolysis time before recovering by reaeration once the current is removed (Figure 7b). In contrast to these dose-proportional responses, the reactive species are considerably self-limiting. Both hydrogen peroxide (Figure 7c) and the ferryl ion (Figure 7e) reach nearly identical peak concentrations irrespective of electrolysis duration, each attaining its maximum at the instant electrolysis stops, declining afterward. This behavior of peroxide and the ferryl ion occurs because Fe­(II) produces and consumes the ferryl ion: dosing more iron speeds up the Fenton reaction, so the extra hydrogen peroxide generated at the cathode is consumed as quickly as it forms, and the oxidant concentration levels off instead of rising with electrolysis time. As a result, doubling the electrolysis time does not increase the peak oxidant concentration; it instead doubles the iron dose, drives the bulk toward oxygen depletion, and shortens the postelectrolysis oxidant lifetime because the enlarged Fe­(II) pool consumes ferryl more rapidly, so that the doubled electrolysis time case exhibits the lowest ferryl concentration beyond about 25 min. These results indicate that where oxidative treatment via the ferryl pathway is the objective, a shorter-to-moderate electrolysis time is preferable. This time-dependent behavior in which oxidant concentration no longer scales with treatment time cannot be reproduced by a model that omits Fe^IV^O^2+^ or treats it as a steady-state quantity and requires the transient approach developed here.

**Figure 7:**
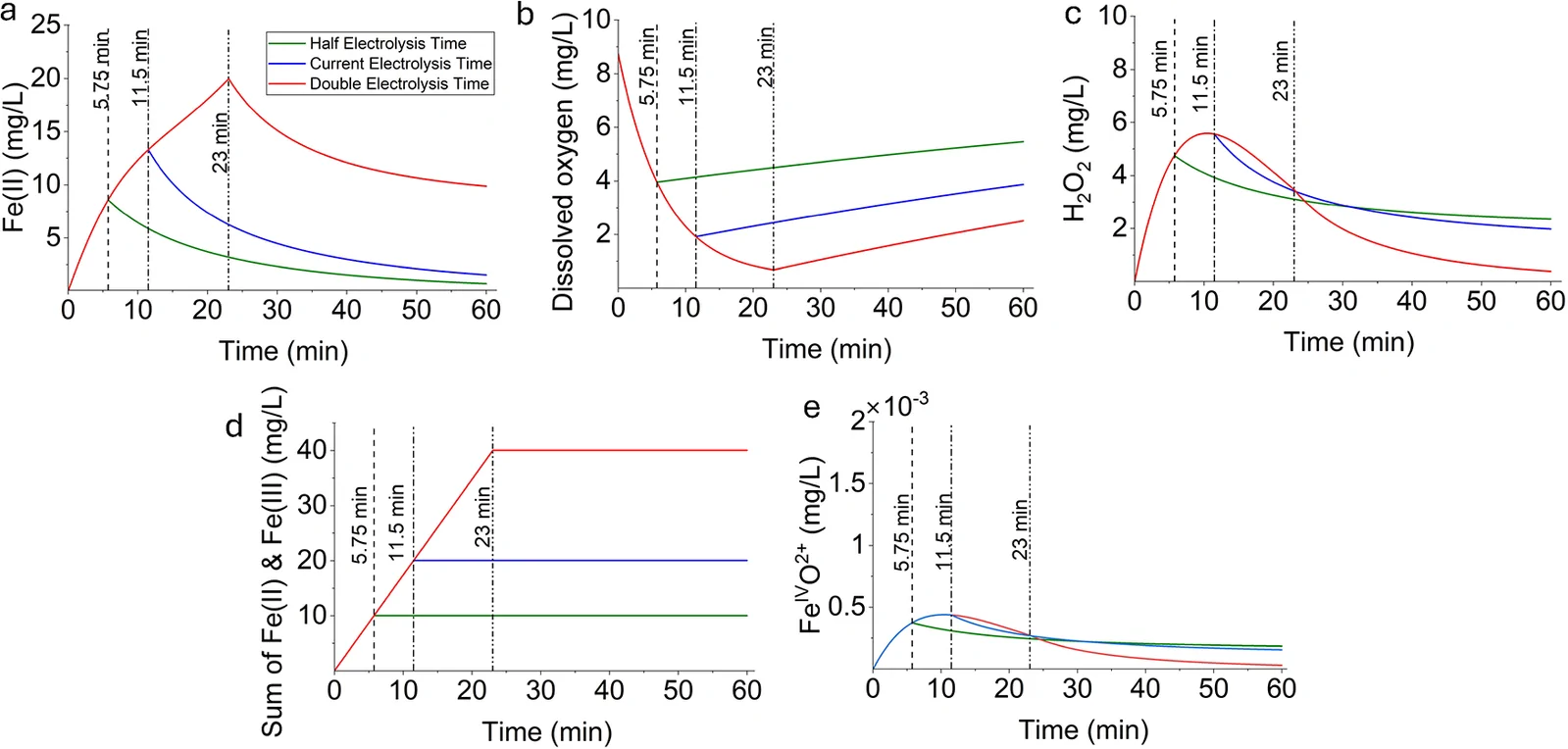
Concentration profiles for half, original, and double the electrolysis times for electrocoagulation at pH 6.5 for the Fe–C cell. Concentrations of (a) ferrous iron (Fe­(II)), (b) dissolved oxygen, (c) hydrogen peroxide (H_2_O_2_), (d) sum of Fe­(II) and Fe­(III), and (e) ferryl ion (Fe^IV^O^2+^).

To evaluate the predictive capability of the model, its performance was tested against an independent experiment conducted at half the original electrolysis time without any fitting (Figure 8a). The parameters obtained from the original fit (Figure 8b) were applied with only the electrolysis duration shortened, and the resulting simulation was compared to the experimental measurements. The model captures the Fe­(II) maximum at the shortened cutoff and its subsequent consumption, the depletion and partial recovery of dissolved oxygen, and the accumulation and decline of hydrogen peroxide, the last of which is somewhat overpredicted at longer times. The level of agreement obtained for the predicted half-electrolysis time condition is comparable to that achieved for the fitted conditions shown in Figure 8b, even though none of the half-electrolysis-time data was used to determine the parameters. This demonstrates that the model’s agreement with experimental data does not stem from tuning parameters separately to each data set but instead reflects a physically sound foundation with parameters that transfer to operating conditions outside the calibration set. The model therefore possesses genuine predictive capability with respect to the measured species, rather than descriptive capability alone.

**Figure 8:**
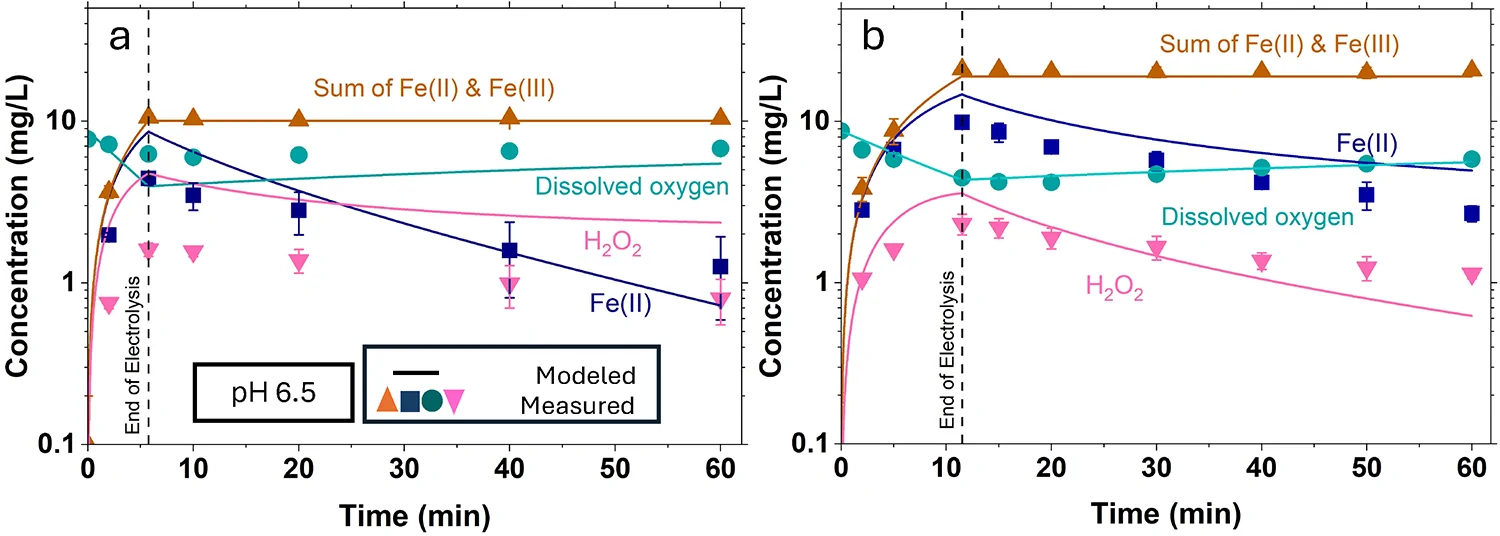
Predictive validation of the kinetic model for the Fe–C configuration at pH 6.5. (a) Model prediction at half the original electrolysis time (5.75 min). (b) Model fit at the current electrolysis time (11.5 min). Solid lines represent modeled profiles and symbols represent measured concentrations.

### 3.6 Discussion

The present model captures the measured concentration profiles of Fe­(II), dissolved oxygen, H_2_O_2_, and total dissolved iron across two electrode configurations and a pH range of 5.0–6.5, providing a consistent description of the coupled interfacial and bulk kinetics, including a prediction of the transient ferryl ion concentration. This model was also used to predict the effects of varying current densities and electrolysis times on the temporal concentrations of Fe­(II), dissolved oxygen, H_2_O_2_, and total dissolved iron. Taken together, these results offer valuable mechanistic insight into electrode identity, operating chemistry, and solution chemistry governing the formation and turnover of reactive species during electrocoagulation.

The experimental data used for this model were collected specifically for this study. Although previous work^34^ reported on pH values of 5.5 and 6.5, this work included two additional pH levels of 5.0 and 6.0 and required all previous experiments to be repeated to obtain the additional sampling time points needed to constrain the model. The experimentally observed Fe­(II), H_2_O_2_, and dissolved oxygen profiles are consistent with previous iron electrocoagulation studies demonstrating anodic Fe­(II) generation and cathodic H_2_O_2_ formation through dissolved oxygen reduction.^34,60,61^ The pronounced dependence of these species on pH also agrees with previous reports showing that Fe­(II) oxidation and subsequent reactive oxidant formation are strongly influenced by solution pH.^54,55,60^ Previous studies demonstrated that the simultaneous presence of Fe­(II) and H_2_O_2_ and the resulting reactive oxidants contribute to contaminant transformation during iron electrocoagulation and related electro-Fenton processes.^57,60,62–64^ However, bulk measurements of Fe­(II), H_2_O_2_, and dissolved oxygen cannot quantitatively resolve the individual reaction pathways or the transient reactive iron species involved. The present kinetic model builds on these experimental findings by quantitatively evaluating the coupled reaction network and the temporal evolution of reactive iron species under the investigated pH conditions and electrode configurations.

However, a limitation inherent to this work is that the ferryl ion cannot be validated via direct experimental measurement due to the ferryl ion’s rapid turnover and low concentration. The ferryl concentration is instead resolved indirectly, and its concentration changes are governed by production via the Fenton reaction and consumption with Fe­(II). The predicted ferryl trajectory should therefore be understood as the profile that is kinetically consistent with the measured species, rather than an independent determination of ferryl concentration. We regard this as an unavoidable consequence of working with a short-lived reactive species and note that the contribution of the present framework is to represent it explicitly and consistently within the model rather than to measure it.

More broadly, the strength of any mechanistic interpretation is bound by the number of species that can be independently measured. The present model accounts for the ferryl ion as the dominant reactive oxygen species on the basis that others such as the hydroxyl radical are minor within the moderate-to-slightly acidic regime studied.^40,41^ However, this assumption was not tested directly, and the relative contributions of competing oxidant pathways, therefore, remain to be resolved experimentally. This is an open question also noted in recent electrocoagulation literature.^34^ The model should thus be regarded as a well-supported but not exhaustively validated representation of the reaction network and applicable at current densities around 9.5–10 A/m^2^, as well as comparable and lower current densities where faradaic efficiency for anodic iron dissolution remains close to 100%, but may not be ideal at substantially higher current densities. Future work should aim to close this gap by incorporating species-resolved measurements of candidate oxidants. Integrating such measurements with the kinetic framework presented here would allow both the predicted ferryl trajectory and assumed negligibility of competing oxidants to be tested directly, transforming the present descriptive model into a fully validated predictive tool for electrocoagulation process design.

We further acknowledge that, because the measured bulk pH varied by less than 0.2 pH units over the course of each experiment, the model treats the proton concentration as constant. Three of the modeled reactions influence solution pH: water reduction and hydrogen peroxide formation at the cathode, and the ferryl ion reduction. All three pH-affecting reactions generate alkalinity, with the cathodic contributions being stoichiometrically bound by the charge passed. The bulk ferryl-reduction is limited by the ferryl concentration, which is predicted to be below 2 μg/L throughout the experiment. This analysis is consistent with experimental measurements where the pH increased no more than 0.2 units, as previously stated, at a charge loading of 66 C/L. and remained marginally elevated for the remainder of the experiment after electrolysis. While adequate for the bulk, this assumption does not capture the local pH changes expected near the electrode surfaces, where cathodic proton consumption and anodic acidification may produce conditions that differ from those in the bulk. Future models should capture dynamic pH evolution and systems operated at higher charge loadings or high coagulant doses.^18^ The model also does not represent Fe­(III) hydrolysis. Fe­(III) simply accumulates in the model and undergoes no further reaction. This is acceptable here because Fe­(III) appears in none of the rate expressions governing the modeled species, and validation is against total iron measured. This simplification would need to be revisited at acidic pH, where hydrolysis is suppressed and free Fe­(III) persists in solution,^18^ allowing cathodic reduction back to Fe­(II);^19^ under those conditions, Fe­(III) would no longer simply accumulate, and the present treatment may not apply.

## 4 Conclusions

Experimental electrocoagulation data were utilized to develop and validate a kinetic model in moderate to slightly acidic media for two different cell configurations. As a result, we estimated the exchange-current density (*i*_o_) and overpotential (η_s_) associated with electrochemical reactions related to the electrochemical dissolution of the anode, water reduction and peroxide formation at the cathode, as well as the volumetric oxygen mass transfer coefficient (*k*_L_ *a*) and the rate constants governing the Fenton reaction responsible for ferryl ion (Fe^IV^O^2+^) formation and consumption.

The predicted ferryl ion (Fe^IV^O^2+^) concentration increased across the mildly acidic to circumneutral pH range in our model. The low predicted ferryl ion concentrations in the bulk electrolyte further suggest that the ferryl ion’s contribution occurs alongside other oxidants within the reaction environments, which together influence the overall extent of contaminant removal. These findings provide a mechanistic basis for understanding how electrocoagulation conditions regulate oxidant formation and reactivity. To date, the relative formation and contributions of Fe^IV^O^2+^ and ^•^OH during iron electrocoagulation have not yet been authoritatively established.^41,42,57,65^ However, previous homogeneous and electro-Fenton studies, including isotopic and probe-based investigations, provided evidence supporting ferryl formation under mildly acidic to near-neutral conditions.^41,42,65–68^ By explicitly incorporating Fe^IV^O^2+^ into the reaction network, the present model provides a mechanistic framework for qualitatively evaluating ferryl ion formation and temporal evolution while remaining constrained by experimentally measurable Fe­(II), H_2_O_2_, and dissolved oxygen concentrations. Thus, rather than assuming Fe^IV^O^2+^ to be the sole reactive oxidant, the model enables evaluation of its potential contribution to iron electrocoagulation chemistry and provides testable predictions for future experimental validations.

Across the conditions tested, our experimental and modeling results indicate that both pH and electrode configuration jointly control the generation of ROS such as H_2_O_2_ and ferryl ion (Fe^IV^O^2+^), which are important species for the removal of wastewater contaminants. By providing a framework to predict ferryl ion concentration, our results give better insight into the lifetime and dynamics of this species at different pH levels under different configurations, which can be used for further control of future electrocoagulation processes. This model also provides a platform for predicting the effects of operating conditions, including varying current density and electrolysis time. The model captures the transient behavior of the oxidant rather than only its end point. Due to Fe^IV^O^2+^ being integrated as a dynamic variable, the model predicts the ferryl ion’s full temporal profile and how the ion evolves with pH and electrode configuration. This provides valuable insight into the detection range required for future experimental work in quantifying the role of the ferryl ion. Future work should also investigate the experimental effects of other pH values, include dynamic changes in pH, and possibly other electrode materials that could further promote the electrochemical formation of ROS. In addition, future model development should incorporate the possibility of dynamic pH evolution (e.g., in cases of low alkalinity and/or high coagulant dosing^69,70^ ), the role of electrode and system parameters (e.g., current density, electrode spacing, electrocoagulant concentrations), and any role of organic matter to better represent electrocoagulation chemistry under more realistic operating conditions.

## Supplementary Material



## Data Availability

The data underlying this study are openly available in FigShare at https://figshare.com/s/d5bb659266ec9fb70e0d.

## Notes

Experimental details and concentration profiles for different applied current densities at various pH values for the two cell configurations (PDF)

## ∥

Department of Environmental and Sustainability Engineering, Indian Institute of Technology Jodhpur, Jodhpur, Rajasthan 342030, India

## Nomenclature

*m_j_*: mass of species *j*
*M_j_*: molecular weight of species *j*
*Q*: charge
*N*: number of electrons per species *j*
*C_j_*: concentrations of species *j*
*F*: Faraday’s constant
*I*: current
*t*: time
*i*: current density
*A*: area
*i*_o_: exchange-current density
α_a_: anodic transfer coefficient
α_c_: cathodic transfer coefficient
*n*: number of electrons transferred
η_s_: surface overpotential
*R*: universal gas constant
*T*: temperature
*C*_ox._: concentration of oxidized species
