# Supplementary material for: Kinetic Modeling of Iron Electrocoagulation in Moderate to Slightly Acidic Media
Source: ACS ES T Water. 2026 Sep 30;6(10):6833–45. doi: 10.1021/acsestwater.6c01166 (PMC13660396; doi:10.1021/acsestwater.6c01166)
Supplement: Supplementary file 1 [file ew6c01166_si_001.pdf]

Supporting Information

Kinetic Modeling of Iron Electrocoagulation in Moderate to Slightly Acidic Media

Edwin I. Ochedikwu[1], Akshat Verma[2], Shankararaman Chellam[2], Benjamin A. Wilhite[1], Jodie Lutkenhaus*[1,3]

[1] Artie McFerrin Department of Chemical Engineering, Texas A&M University, TX 77843, United States of America

[2]Department of Civil & Environmental Engineering, Texas A&M University, TX 77843, United States of America

[3] Department of Materials Science and Engineering, Texas A&M University, TX 77840, United States of America

Experimental details

Electrocoagulation tests were conducted in a 500 mL stirred batch reactor using a synthetic solution prepared to represent the inorganic chemistry of municipal secondary wastewater effluents[1, 2] (i.e., influent to potable water treatment plants). The matrix had ~300 mg/L as $CaCO_3$ total hardness, ~100 mg/L as $CaCO_3$ alkalinity, chloride (300 mg/L), sulfate (200 mg/L), and silicate (40 mg/L). Organic matter was not included in order to avoid analytical interference and reactions with short-lived oxidants during electrochemical treatment, similar to our other publications.[3, 4] The complete ionic composition and preparation details are provided in **Supporting Information (SI) Table S1**.

Triplicate experiments were conducted for 60 minutes at a total iron dose of 20 mg/L and pH values of 5.0, 5.5, 6.0, and 6.5 using two electrode configurations: (i) identical ground low-carbon steel plate anode and cathode (~99% Fe; McMaster-Carr, catalog number 1388K454, **SI Table S2**) termed "Fe–Fe electrocoagulation" and (ii) ground low-carbon steel plate anode and graphite cathode (100% C; McMaster-Carr, 9121K61), termed "Fe–C electrocoagulation". All experimental data reported in this work is new. Experiments at pH 5.0 and 6.0 have not been previously reported and although pH 5.5 and 6.5 have been examined in previous work[3], the data presented here came from experiments performed specifically for this work. The selected pH range was chosen to encompass conditions previously associated with enhanced virus control during iron electrocoagulation while also enabling evaluation of pH-dependent oxidant formation within the mildly acidic to near-neutral region where ferryl-mediated Fenton chemistry becomes increasingly important.[5-8] The solution pH was adjusted to the desired initial value using HCl prior to electrocoagulation and subsequently monitored throughout the experiment. The adjusted solution pH remained within 0.2 pH units of the target value throughout the experiments.

The electrode wetted surface area was 50 cm^2, separation distance was 1 cm, and current density was held constant at 0.95 mA/cm^2 using a regulated power supply (Interface 1010E, Gamry Instruments). These operational parameters, including the inter-electrode spacing (1 cm), electrode area, current density, and target iron dose, were adopted from our previous extensive work on electrocoagulation protocols.[3, 4, 9, 10] We emphasize that EC performance including reactive oxygen species (ROS) production will depend on these experimental conditions, which were not varied during this study. The suspension was rapidly mixed at 350 rpm (coagulation) during the entire 11.5 minutes of electrolysis and followed with 48.5 minutes of slow mixing at 50 rpm (flocculation). Minimal iron dissolution (0.2-0.4 mg/L) was observed in the absence of current across all pH conditions. Additional details are in **SI Sections S1**.

Concentrations of total Fe, Fe(II), and H_2O_2, and pH and dissolved oxygen were monitored (see **SI Section S2**).

Table S1. Ionic composition of the synthetic solution used in this study.

**Parameters**	**Concentration**	
Analytes	**mg/L**	**mM**
Ca^{2+}	75	1.9
Mg^{2+}	25	1.0
Na^{+}	225	9.8
SiO_3^{2-}	38	0.5
Cl^{-}	300	8.4
SO_4^{2-}	200	2.1
HCO_3^{-}	122	61
Hardness (as $CaCO_3$)	292	2.9
Alkalinity (as $CaCO_3$)$^{\alpha}$	~100	0.1
General parameters *(units in paratheses)*		
pH (no unit)$^{\beta}$	5.0, 5.5, 6.0, 6.5	
Conductivity (µS/cm)	1550	

[α] concentrations depend on the pH values

[β] The solution pH was adjusted to the desired initial value using HCl prior to electrocoagulation and was subsequently monitored throughout the experiment. Continuous feedback control was not employed.

Table S2. Elemental composition of the electrodes used in this study (wt%).

Element	Iron electrode (carbon steel)	Conductive graphite electrode
Fe	>98.9	-
C	<0.25	100
Cu	>0.20	-
P	<0.03	-
Si	<0.40	-
S	<0.03	-

[a] Link for Fe (carbon steel) electrode: https://www.mcmaster.com/1388K454/

[b] Link for C (graphite) electrode: https://www.mcmaster.com/9121K61/

Section S1. Additional operational details and oxygen reduction pathways

Electrodes were regenerated between successive electrocoagulation runs to maintain consistent surface conditions. Deposited iron layers were removed by rinsing the plates with acidified water (pH ~3) followed by washing with deionized water. Mechanical polishing was then carried out sequentially using 120- and 220-grit sandpapers, after which the electrodes were thoroughly rinsed with deionized water prior to reuse. During electrocoagulation, the cell potential was continuously monitored.

Hydrogen peroxide **(**H_2O_2**)** formation at the cathode arises from electrochemical reduction of dissolved oxygen.[11] Oxygen reduction may proceed through either a four-electron pathway producing water or a two-electron pathway producing H_2O_2, represented as:[11, 12]

$$O_2 + 4e^- + 4H^+ \rightarrow 2H_2O \quad \text{S3}$$

$$O_2 + 2e^- + 4H^+ \rightarrow 2H_2O_2 \quad \text{S4}$$

Both processes involve formation of surface-bound oxygen intermediates. A simplified representation of the reaction sequence is:

$$O_2 + 4e^- + 4H^+ \rightarrow *OOH + 3(H^+ + e^-) \rightarrow *O + 2(H^+ + e^-) \rightarrow *OH + 1(H^+ + e^-) \rightarrow 2H_2O \quad \text{S5}$$

$$O_2 + 2e^- + 2H^+ \rightarrow *OOH + (H^+ + e^-) \rightarrow H_2O_2 \quad \text{S6}$$

The hydroperoxyl intermediate (*OOH) is common to both pathways. The relative preference for the four- or two-electron route depends on the interaction between this intermediate and the electrode surface.[11, 12] Strong adsorption favors further reduction toward water, whereas weaker adsorption promotes release of hydrogen peroxide.[11] Carbon (graphitic) cathodes generally exhibit weaker adsorption of oxygen intermediates, which increases selectivity toward the two-electron oxygen reduction pathway and H_2O_2 generation.[11]

Section S2. Analytical measurements

Several aqueous parameters were measured during electrocoagulation to evaluate changes in solution composition. pH**,** dissolved oxygen (DO), and electrical conductivity were monitored in the bulk solution using an Orion Basic pH meter (Thermo Fisher Scientific), a luminescent dissolved oxygen probe (LDO101, HACH), and a conductivity probe (CDC401, HACH), respectively.[3, 13, 14]

The concentration of total dissolved iron was determined using the colorimetric HACH Method 8112.[15, 16] In this assay, ferric iron present in the sample is chemically converted to ferrous iron using sodium metabisulfite. The ferrous ions then react with 2,4,6-tripyridyl-s-triazine (TPTZ) to produce a colored Fe–TPTZ complex. The intensity of the resulting purple complex was quantified spectrophotometrically at 590 nm using a HACH DR6000 spectrophotometer, and concentrations were obtained according to the method calibration.

Ferrous iron (Fe(II)) concentrations were measured using HACH Method 8146, which relies on the formation of a colored complex between Fe(II) and 1,10-phenanthroline.[16] The pink complex formed in this reaction was measured by absorbance at 510 nm with the same spectrophotometer, and Fe(II) levels were calculated following the prescribed analytical protocol. The concentration of ferric iron (Fe(III)) was not measured directly but was determined by finding the difference between total dissolved iron and Fe(II) concentration measurements.

The concentration of hydrogen peroxide (H_2O_2) was quantified using the Cu(I)–2,9-dimethyl-1,10-phenanthroline colorimetric method.[17, 18] In this procedure, hydrogen peroxide participates in a redox reaction involving copper, leading to formation of an orange-colored Cu–phenanthroline complex that can be measured spectrophotometrically at 454 nm. Prior to analysis, samples were passed through 0.2 μm membrane filters to remove suspended material. To eliminate potential

interference caused by Fe(II)-driven reduction of Cu(II) during the assay, 7.2 mM sodium citrate was added to the filtered samples as a complexing agent.[14]

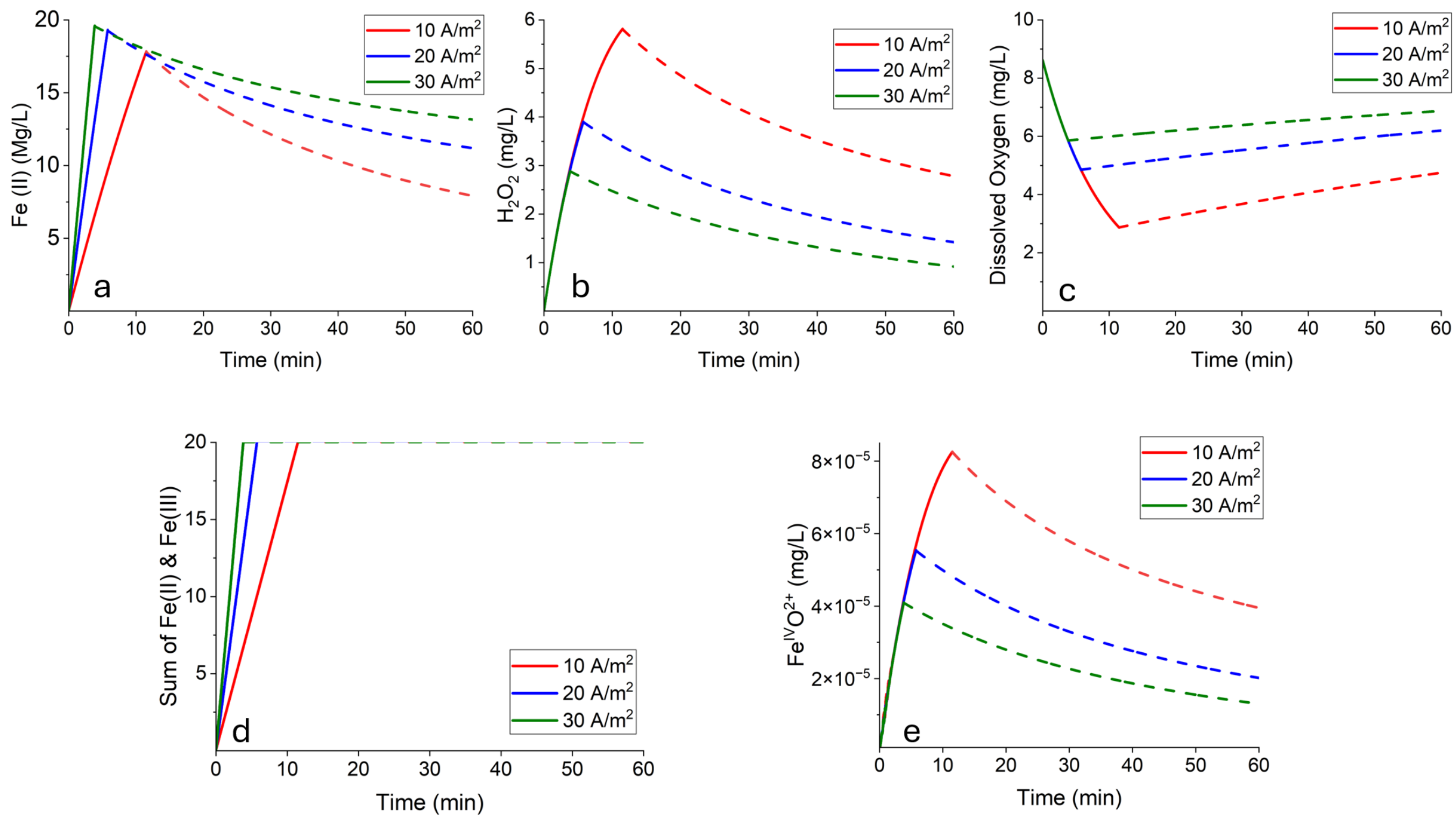

Figure S1. Concentration profiles for different applied current densities at *pH 5.0* for the *Fe-C configuration*. (a) Ferrous iron (Fe(II)), (b) hydrogen peroxide (H_2O_2), (c) dissolved oxygen, (d) sum of Fe(II) and Fe (III), ferryl ion ($Fe^{IV}O^{2+}$). The solid lines represent the electrolysis period while the dotted lines represent the time after electrolysis.

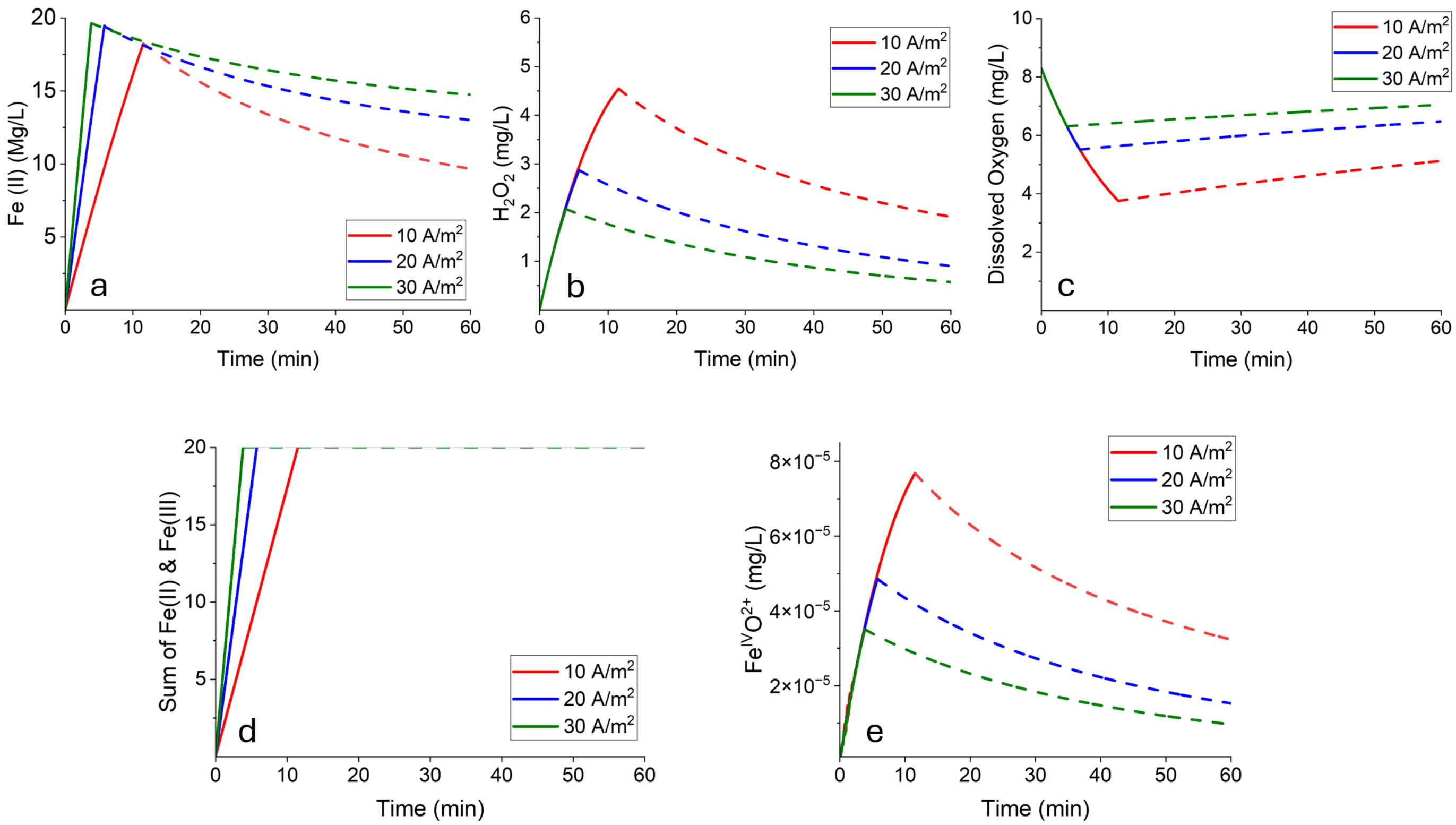

Figure S2 Concentration profiles for different applied current densities at *pH 5.5* for the Fe-C *configuration*. (a) Ferrous iron (Fe(II)), (b) hydrogen peroxide (H_2O_2), (c) dissolved oxygen, (d) sum of Fe(II) and Fe (III), ferryl ion ($Fe^{IV}O^{2+}$). The solid lines represent the electrolysis period while the dotted lines represent the time after electrolysis.

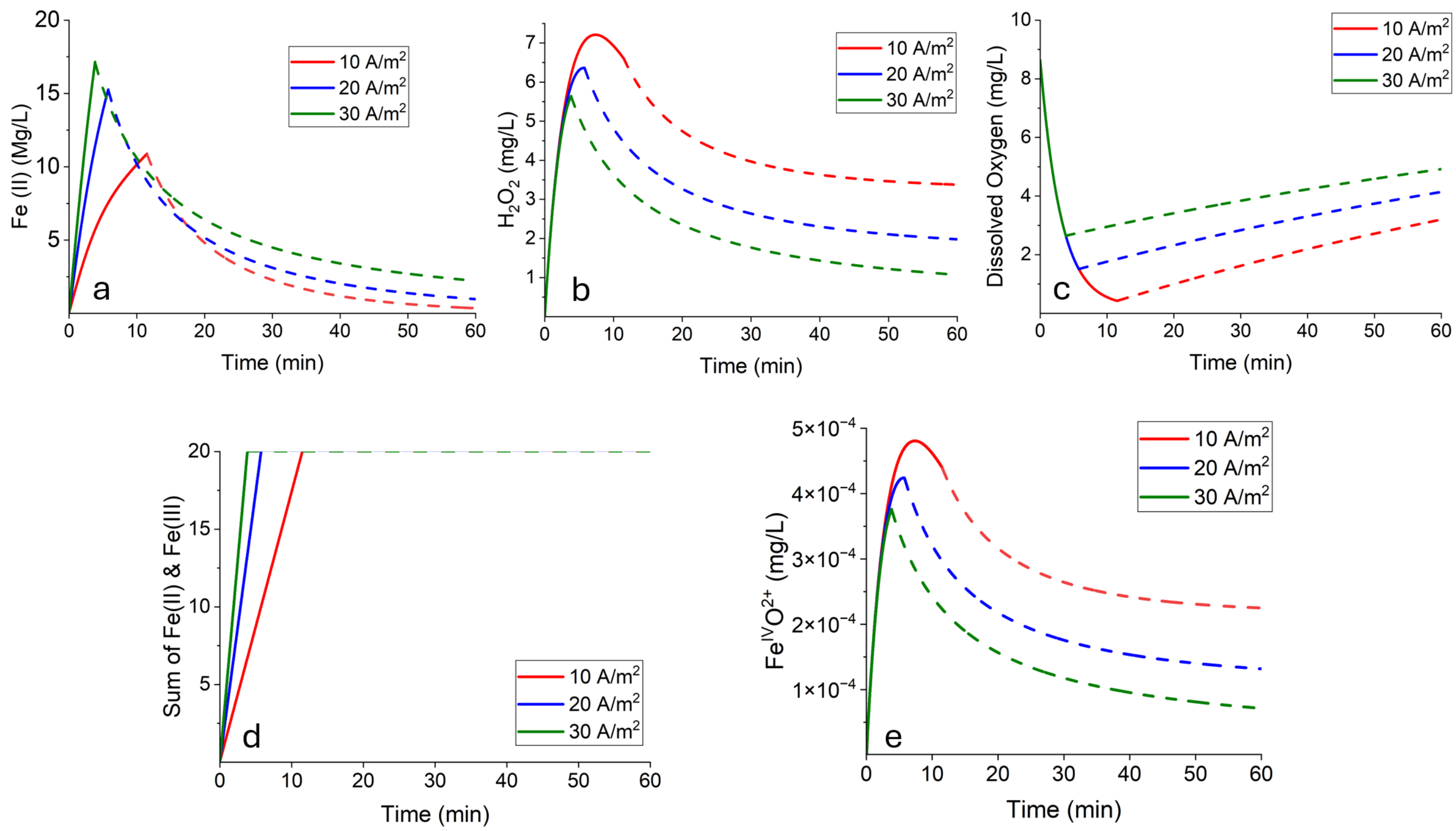

Figure S3 Concentration profiles for different applied current densities at *pH 6.0* for the Fe-C *configuration*. (a) Ferrous iron (Fe(II)), (b) hydrogen peroxide (H_2O_2), (c) dissolved oxygen, (d) sum of Fe(II) and Fe (III), ferryl ion ($Fe^{IV}O^{2+}$). The solid lines represent the electrolysis period while the dotted lines represent the time after electrolysis.

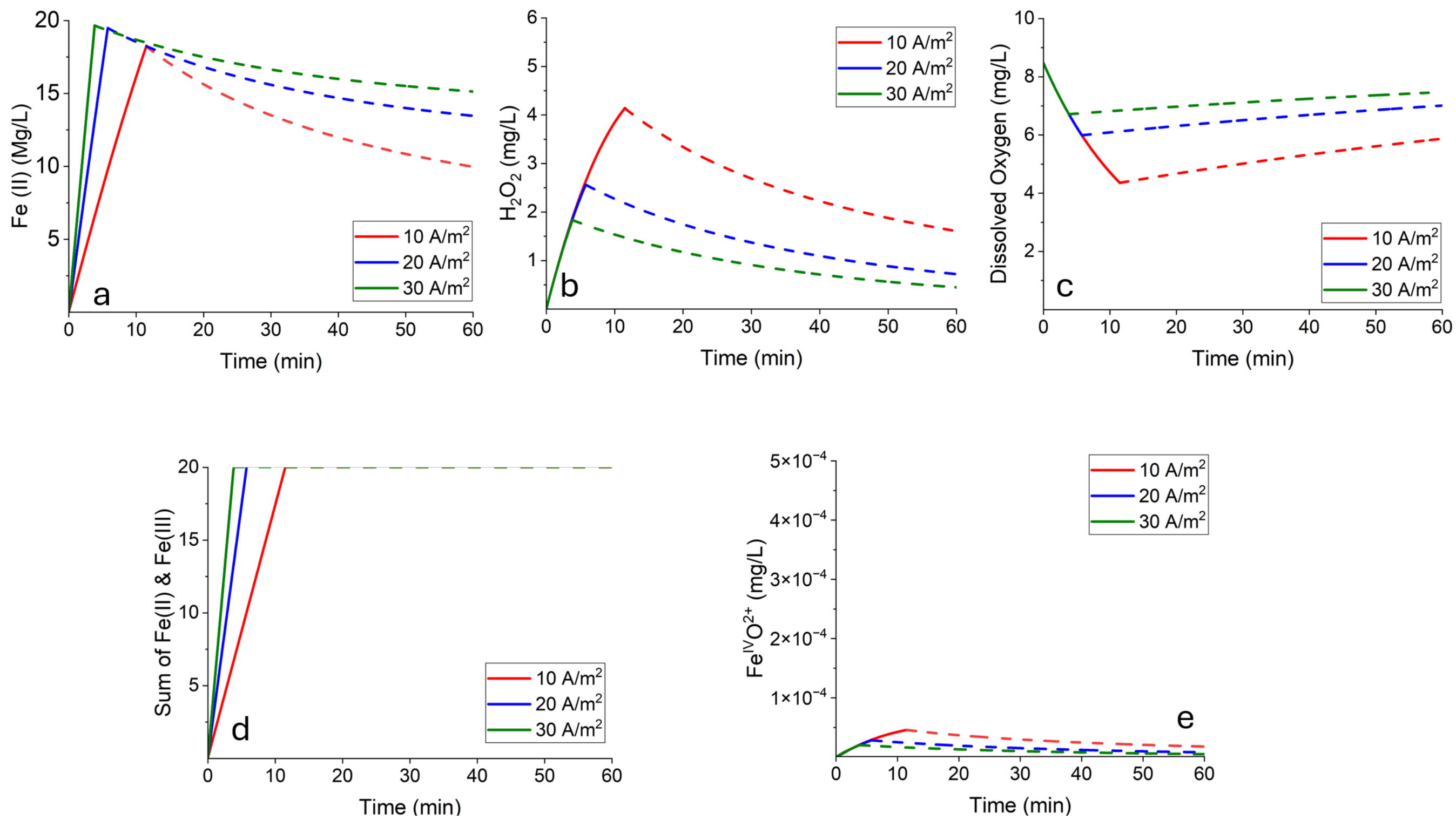

Figure S4. Concentration profiles for different applied current densities at *pH 5.0* for the *Fe-Fe configuration*. (a) Ferrous iron (Fe(II)), (b) hydrogen peroxide (H_2O_2), (c) dissolved oxygen, (d) sum of Fe(II) and Fe (III), ferryl ion ($Fe^{IV}O^{2+}$). The solid lines represent the electrolysis period while the dotted lines represent the time after electrolysis.

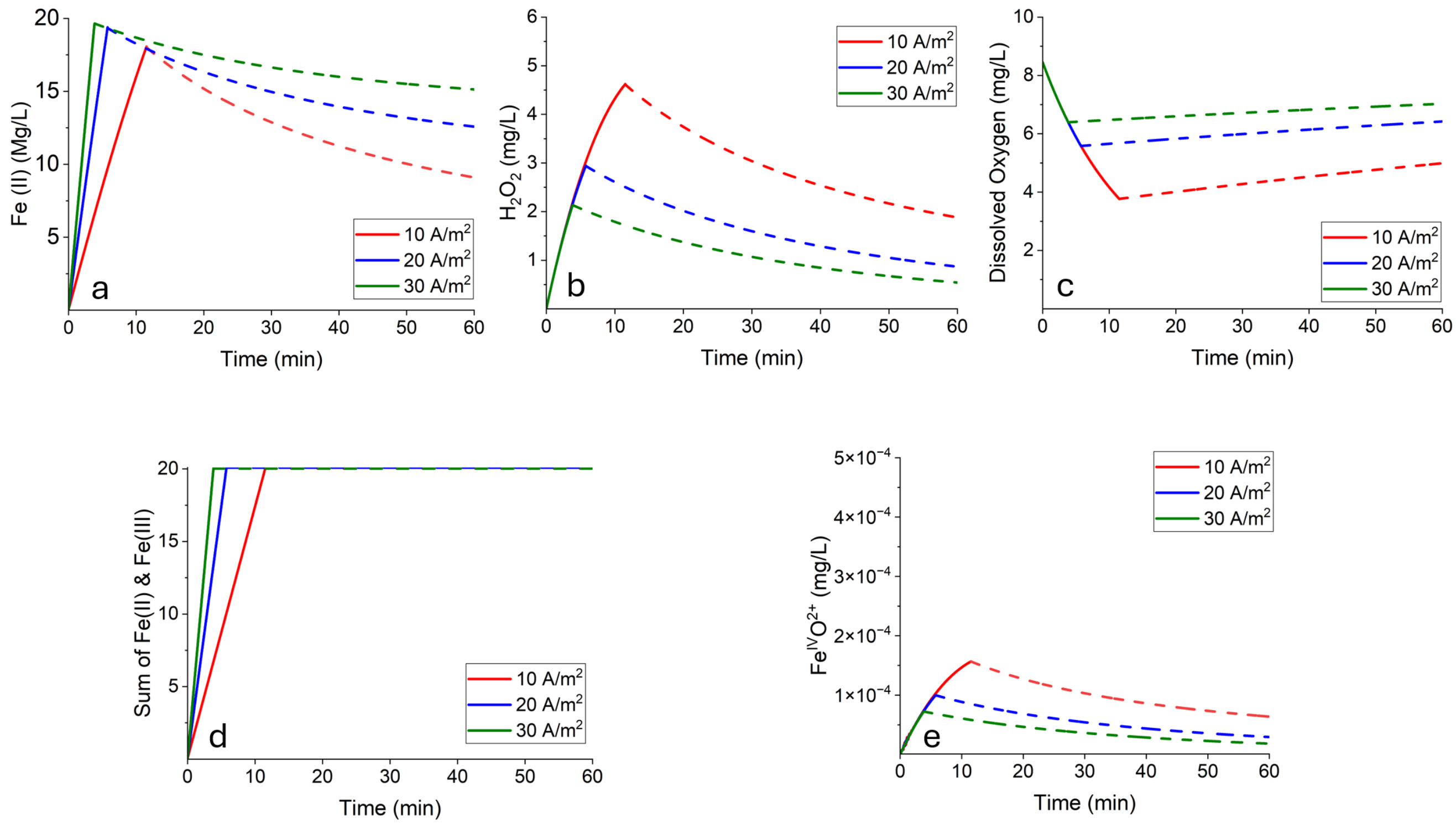

Figure S5. Concentration profiles for different applied current densities at *pH 5.5* for the *Fe-Fe configuration*. (a) Ferrous iron (Fe(II)), (b) hydrogen peroxide (H_2O_2), (c) dissolved oxygen, (d) sum of Fe(II) and Fe (III), ferryl ion ($Fe^{IV}O^{2+}$). The solid lines represent the electrolysis period while the dotted lines represent the time after electrolysis.

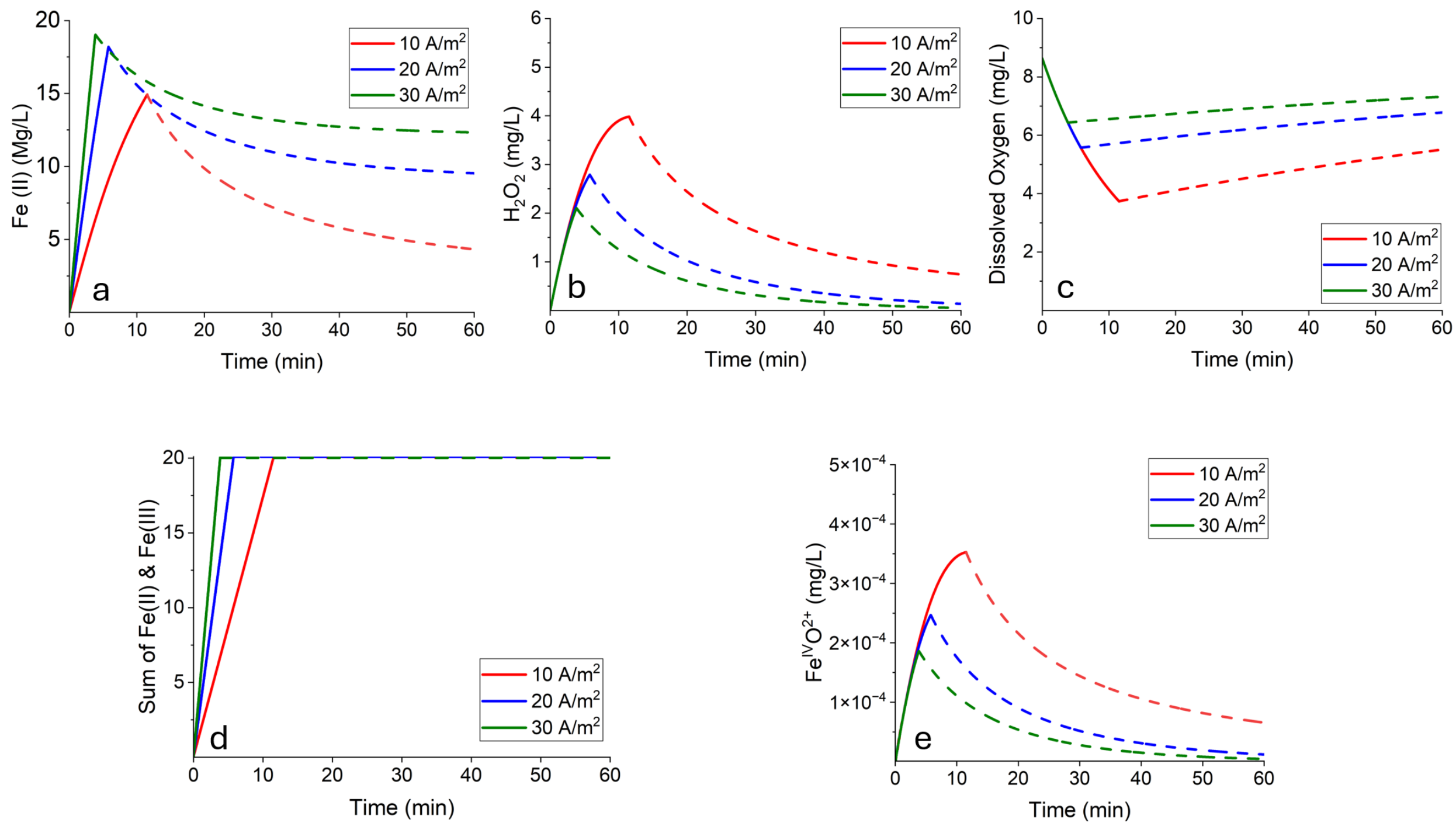

Figure S6. Concentration profiles for different applied current densities at *pH 6.0* for the *Fe-Fe configuration*. (a) Ferrous iron (Fe(II)), (b) hydrogen peroxide (H_2O_2), (c) dissolved oxygen, (d) sum of Fe(II) and Fe (III), ferryl ion ($Fe^{IV}O^{2+}$). The solid lines represent the electrolysis period while the dotted lines represent the time after electrolysis.

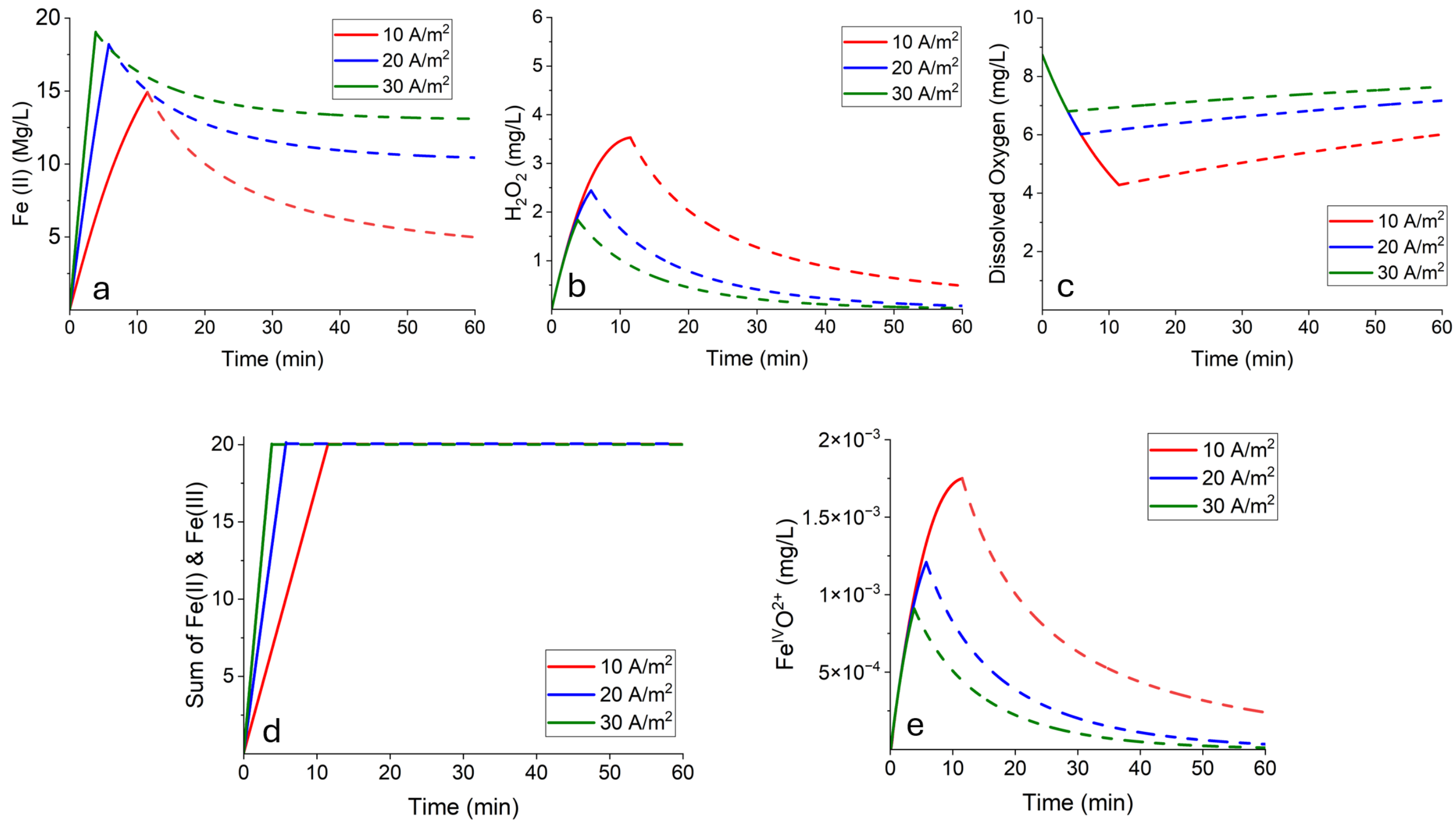

Figure S7. Concentration profiles for different applied current densities at *pH 6.5* for the *Fe-Fe configuration*. (a) Ferrous iron (Fe(II)), (b) hydrogen peroxide (H_2O_2), (c) dissolved oxygen, (d) sum of Fe(II) and Fe (III), ferryl ion ($Fe^{IV}O^{2+}$). The solid lines represent the electrolysis period while the dotted lines represent the time after electrolysis.

References

(1) Abada, B.; Joag, S.; Alspach, B.; Bustamante, A.; Chellam, S. Inorganic and Organic Silicon Fouling of Nanofiltration Membranes during Pilot-Scale Direct Potable Reuse. *ACS ES&T Engineering* **2023**, *3* (9), 1413–1423. DOI: 10.1021/acsestengg.3c00172.

(2) Abada, B.; Safarik, J.; Ishida, K. P.; Chellam, S. Elucidating Foulant Diversity during Full-Scale Potable Reuse: Forensic Analysis of Lead and Lag Elements of a Three-Stage Reverse Osmosis System. *ACS ES&T Engineering* **2022**, *2* (11), 2116–2129. DOI: 10.1021/acsestengg.2c00171.

(3) Verma, A.; Chellam, S. Virus Capsid Modifications Accompanying Inactivation during Iron Electrocoagulation Revealed by Proteomics, Infrared Spectroscopy, and Molecular Modeling. *Environmental Science & Technology* **2025**. DOI: 10.1021/acs.est.5c13277.

(4) Kim, K.; Castillo, C.; Jang, G. G.; Zhang, Y.; Tsouris, C.; Chellam, S. Porous Iron Electrodes Reduce Energy Consumption During Electrocoagulation of a Virus Surrogate: Insights into Performance Enhancements Using Three-Dimensional Neutron Computed Tomography. *ACS Es&t Engineering* **2024**, *4* (10), 2573–2584.

(5) Kim, J. Y.; Lee, C.; Sedlak, D. L.; Yoon, J.; Nelson, K. L. Inactivation of MS2 coliphage by Fenton's reagent. *Water Research* **2010**, *44* (8), 2647–2653. DOI: https://doi.org/10.1016/j.watres.2010.01.025.

(6) Deng, G.; Wang, Z.; Ma, J.; Jiang, J.; He, D.; Li, X.; Szczuka, A.; Zhang, Z. Ferryl ion in the photo-fenton process at acidic pH: occurrence, fate, and implications. *Environmental Science & Technology* **2023**, *57* (47), 18586–18596.

(7) Bataineh, H.; Pestovsky, O.; Bakac, A. pH-induced mechanistic changeover from hydroxyl radicals to iron (IV) in the Fenton reaction. *Chemical Science* **2012**, *3* (5), 1594–1599.

(8) Koppenol, W. H. Ferryl for real. The Fenton reaction near neutral pH. *Dalton Transactions* **2022**, *51* (45), 17496–17502.

(9) Kim, K.; Sen, A.; Chellam, S. Facile Control of a Tailed Virus Surrogate by Iron Conventional Coagulation and Electrocoagulation. *Environmental Science & Technology* **2025**, *59* (31), 16697–16708. DOI: 10.1021/acs.est.5c02792.

(10) Kim, K.; Sen, A.; Chellam, S. Removal and inactivation of nonenveloped and enveloped virus surrogates by conventional coagulation and electrocoagulation using aluminum and iron. *ACS ES&T Engineering* **2022**, *2* (10), 1974–1986.

(11) Wang, N.; Ma, S.; Zuo, P.; Duan, J.; Hou, B. Recent Progress of Electrochemical Production of Hydrogen Peroxide by Two-Electron Oxygen Reduction Reaction. *Advanced Science* **2021**, *8* (15), 2100076. DOI: https://doi.org/10.1002/advs.202100076 (accessed 2026/03/25).

(12) Kulkarni, A.; Siahrostami, S.; Patel, A.; Nørskov, J. K. Understanding Catalytic Activity Trends in the Oxygen Reduction Reaction. *Chemical Reviews* **2018**, *118* (5), 2302–2312. DOI: 10.1021/acs.chemrev.7b00488.

(13) Kim, K.; Jothikumar, N.; Sen, A.; Murphy, J. L.; Chellam, S. Removal and Inactivation of an Enveloped Virus Surrogate by Iron Conventional Coagulation and Electrocoagulation. *Environmental Science & Technology* **2021**, *55* (4), 2674–2683. DOI: 10.1021/acs.est.0c07697.

(14) Kim, K.; Narayanan, J.; Sen, A.; Chellam, S. Virus Removal and Inactivation Mechanisms during Iron Electrocoagulation: Capsid and Genome Damages and Electro-Fenton Reactions. *Environmental Science & Technology* **2021**, *55* (19), 13198–13208. DOI: 10.1021/acs.est.0c04438.
(15) Stookey, L. L. Ferrozine---a new spectrophotometric reagent for iron. *Analytical Chemistry* **1970**, *42* (7), 779–781. DOI: 10.1021/ac60289a016.
(16) PART 9000 MICROBIOLOGICAL ANALYSIS OF WATER AND WASTEWATER. In *Standard Methods For the Examination of Water and Wastewater*, Standard Methods for the Examination of Water and Wastewater, American Public Health Association, 2017.
(17) Florence, T. M.; Stauber, J. L.; Mann, K. J. The reaction of copper-2,9-dimethyl-1,10-phenanthroline with hydrogen peroxide. *Journal of Inorganic Biochemistry* **1985**, *24* (4), 243–254. DOI: https://doi.org/10.1016/0162-0134(85)85053-4.
(18) Baga, A. N.; Johnson, G. R. A.; Nazhat, N. B.; Saadalla-Nazhat, R. A. A simple spectrophotometric determination of hydrogen peroxide at low concentrations in aqueous solution. *Analytica Chimica Acta* **1988**, *204*, 349–353. DOI: https://doi.org/10.1016/S0003-2670(00)86374-6.
